# Closing the screening gap but not the writing gap: a two-topic evaluation of LLMs for systematic reviews and meta-analyses in hepatology

**DOI:** 10.1038/s44355-026-00068-w

**Published:** 2026-06-22

**Authors:** Yuntao Zou, Iris Kim, Nan Gao, Michelle Li, Mi-Ok Kim, Jin Ge

**Affiliations:** 1 University of California San Francisco, Department of Medicine, Division of Hospital Medicine, San Francisco, CA USA; 2 University of California San Francisco, Department of Epidemiology and Biostatistics, San Francisco, CA USA; 3 Montefiore Medical Center, Bronx, NY USA; 4 University of California San Francisco, Department of Medicine, Division of Gastroenterology and Hepatology, San Francisco, CA USA

**Keywords:** Diseases, Health care, Medical research

## Abstract

Systematic reviews are essential but labor-intensive. We evaluated LLM-assisted literature screening and drafting in two hepatology topics: carvedilol in compensated cirrhosis and anticoagulation in portal vein thrombosis. For each topic, we searched PubMed, Cochrane, and EMBASE. A few-shot prompt with explicit inclusion/exclusion criteria was used to screen titles and abstracts, with results compared to manual review. Included studies were then processed using a retrieval-augmented LLM to generate ten automated systematic review and meta-analysis drafts per topic, which were evaluated by a separate judge LLM for PRISMA 2020 compliance against human reviews. Screening performance: After deduplication (703 and 370 records), LLM-assisted screening showed high agreement with manual review (sensitivity 86–93%, specificity 96–99%) while reducing screening time to 3 and 2 h versus 62 and 30 h manually. Drafting performance: RAG-enabled LLMs generated structured manuscripts with variable PRISMA 2020 compliance: 100% for titles, 91% for introductions, 75–80% for methods, and 68–75% for results, with downstream weaknesses in abstracts and discussions (<65%). LLM-based PRISMA scoring closely matched human review (ICC ≈ 0.90). LLM-assisted screening was highly accurate, reducing workload by >90%, but automated drafting was reliable mainly for titles and introductions, requiring human oversight to prevent errors and hallucinations.

## Introduction

Systematic reviews and meta‑analyses underpin evidence‑based hepatology by synthesizing data from multiple studies^1^. As clinical research output in liver diseases accelerates, researchers face mounting challenges in identifying and screening relevant studies for evidence synthesis^2,3^. In the construction of systematic reviews and meta-analyses, the article‑review stage (e.g., especially title/abstract screening) is especially time‑intensive. Researchers must navigate heterogeneous study designs while applying domain‑specific inclusion/exclusion criteria. Manual screening workflows adhering to Preferred Reporting Items for Systematic Reviews and Meta-Analyses (PRISMA) guidelines can take weeks to months^4^. Moreover, manual human screening is also vulnerable to inter‑reviewer variability and errors, which are often dependent upon the expertise level of the review.

To reduce this bottleneck, researchers have begun adopting semi‑automated and automated tools, such as machine learning (ML) classifiers, text‑mining, and rule‑based filters, to triage manuscripts and prioritize relevant records for human review^5–8^. Developing these tools, however, requires curated domain‑specific training data or rule sets, and their performance can degrade when applied to specific clinical topics, such as those within hepatology. More importantly, most existing tools are narrowly focused on screening and do not extend to downstream stages of evidence synthesis.

Beyond screening, the drafting and synthesis phase of systematic reviews and meta-analyses constitutes an equally substantial, but less frequently discussed, bottleneck. Constructing PRISMA-compliant manuscripts requires integrating heterogeneous evidence into coherent narrative sections, accurately contextualizing quantitative results, and maintaining consistency across methods, results, and discussion. This process demands substantial domain expertise and methodological fluency, and it is a major contributor to the time, cost, and limited scalability of high-quality evidence synthesis. Despite advances in screening automation, few computational approaches have attempted to support this higher-order task of structured scientific synthesis.

Recent advances in large language models (LLMs) offer a new opportunity to address both challenges. Unlike traditional classifiers, LLMs can perform context-aware reasoning over unstructured biomedical text and generate structured outputs that resemble human scientific writing. Prior work in other biomedical domains has shown that LLMs can improve efficiency and consistency in abstract screening task^9,10^. Their potential role in drafting systematic review and meta-analysis manuscripts, while adhering to established reporting standards, however, remains largely unexplored, particularly within specialty-specific domains such as hepatology.

Given these opportunities and risks posed by LLMs, we sought to demonstrate the potential of LLMs in aiding the construction of systemic reviews and meta-analyses in hepatology. In this study, we investigated the feasibility, accuracy, and efficiency of employing LLMs for two tasks:

1. Title and abstract screening within systematic reviews and meta-analyses—in this task, we compared LLM-assisted screening versus manual human review and screening (in terms of sensitivity, specificity, and workload reduction).
2. Drafting “synthetic” systematic review and meta-analysis manuscripts—in this task, we constructed a customized LLM implementation through Retrieval Augmented Generation (RAG) using the articles selected by the screener. We then used prompt engineering to ask the customized RAG-LLM to draft portions of a systematic review and meta-analysis manuscript. To evaluate the quality of the “synthetic” drafts, we employed an “LLM-as-a-judge” framework to systematically and reproducibly evaluate the quality against the PRISMA evaluation framework^11^.

For our motivating studies, we sought to emulate and replicate two seminal systematic reviews and meta-analyses published within hepatology and liver diseases regarding the following clinical topics:

1. Whether carvedilol reduces decompensation and mortality in patients with compensated cirrhosis^12^?
2. Whether anticoagulant therapy has an impact on portal vein thrombosis (PVT) recanalization and progression in patients with cirrhosis^13^?

## Results

### Topic 1—carvedilol in compensated cirrhosis

A total of 1077 records were retrieved from multiple bibliographic databases. Upon initial processing, 374 records (35%) were identified as duplicates based on matching titles, Digital Object Identifiers (DOIs), and other metadata elements. After removal of these duplicates, 703 unique records remained for the subsequent screening phase. Compared to manual review as the reference standard, the LLM achieved a sensitivity of 93% (95% confidence interval [CI]: 66–100%) and a specificity of 96% (95% CI: 94–97%) for the 703 unique records. False negatives (~7%) primarily involved abstracts with nuanced methodological details or complex population criteria not clearly described in the abstract text. False positives (~4%) occurred when abstracts contained relevant keywords but failed to meet one or more inclusion criteria upon full-text assessment.

As a sensitivity analysis, we also applied the LLM-assisted screening pipeline to all 1077 records (including duplicates), instructing the LLM first to flag duplicates and subsequently to apply inclusion/exclusion decisions. Performance metrics were similar to those obtained on the deduplicated set: sensitivity 93% (95% CI: 66–100%) and specificity 95% (95% CI: 93–96%). The LLM-assisted screening of all abstracts required approximately 3 h of processing time and consumed around 30 million tokens, corresponding to an estimated cost of USD$82.50. In contrast, manual screening was estimated to require approximately 62 h of reviewer time at approximately 3.5 min per article.

Across the ten “synthetic” manuscripts generated, the LLM “judge” showed that “Title” sections achieved perfect scores, making it the highest-performing category (Table 1). The “Introduction” section followed with an average of 91%, while the “Methods” section had an average score of 74%. The “Results” sections averaged 68%, its main weakness stemming from inaccurate extraction and synthesis of numerical data from full-text PDFs, including misreported sample sizes, effect estimates, and confidence intervals, as well as omission of key outcomes. In several cases, the model generated plausible but nonexistent findings (e.g., hallucinations). Performances were lower in the “Abstract,” “Discussion,” and “Others” sections, which averaged 20–60% of full potential section scores under the PRISMA 2020 checklist. As the “Results” sections of these “synthetic” manuscripts were flawed, the interpretation of the “Discussion,” “Abstract,” and “Other” section scores is unreliable.

**Table 1:** Manuscript drafting performance for carvedilol in compensated cirrhosis

Section	Average score (% of full)	Mean ± SD (raw score)	Relative rank
Title	100%	1.00 (all full)	Highest
Introduction	91.25%	1.83 ± 0.17	2nd
Methods	73.82%	12.55 ± 1.87	3rd
Results	67.62%	7.44 ± 2.35	4th
Abstract	59.10%	7.09 ± 1.87	5th
Discussion	51.56%	2.06 ± 0.66	6th
Other	28.44%	1.71 ± 1.27	Lowest

### Topic 2—anticoagulation in PVT

A total of 464 records were retrieved from multiple bibliographic databases. Upon initial processing, 94 records (20%) were identified as duplicates based on matching titles, DOIs, and other metadata elements. After removal of these duplicates, 370 unique records remained for the subsequent screening phase. LLM-assisted screening was compared to manual review as the reference standard for the 370 unique records. The LLM achieved a sensitivity of 86% (95% CI: 65–97%) and a specificity of 99% (95% CI: 98–100%). False negatives (approximately 1%) similarly mostly occurred in abstracts where nuanced methodological details or complex population criteria were not clearly described. False positives (approximately 1%) occurred when abstracts contained relevant keywords but failed to meet all inclusion criteria. In these cases, the LLM falsely ruled in articles addressing primary PVT prevention rather than the impact on existing PVT.

As a sensitivity analysis, we also applied the LLM-assisted screening pipeline to all 464 records (including duplicates), instructing the LLM first to flag duplicates and subsequently to apply inclusion/exclusion decisions. Performance metrics were similar to those obtained on the deduplicated set: sensitivity 82% (95% CI: 57–96%) and specificity 99% (95% CI: 99–100%). LLM-assisted screening of all abstracts required approximately 2 h of processing time and consumed around 14 million tokens, corresponding to an estimated cost of USD $38.40. In contrast, manual screening was estimated to require approximately 30 h of reviewer time (approximately 3.9 min per article).

Across the ten “synthetic” manuscripts generated, the “LLM” judge again showed that the “Title” sections had perfect scores, ranking as the strongest performer (Table 2). The “Introduction” section had an average score of 91% while the “Methods” section had an 80% average. The “Results” sections average 75%. Again, there were persistent problems with the extraction and synthesis of numerical data from the originating manuscripts. The “Abstract” sections averaged 55%, reflecting weaker content compared to the core sections. The “Discussion” sections averaged 65%, but the substantive interpretations were undermined by reliance on inaccurate or nonexistent results. The “Other” Section remained the weakest, averaging only 16%, with key elements such as data availability, conflicts of interest, and funding disclosures often missing or poorly described.

**Table 2:** Manuscript drafting performance for anticoagulation in PVT

Section	Average score (% of full)	Mean ± SD (raw score)	Relative rank
Title	100%	1.00 (all full)	Highest
Introduction	91.00%	1.82 ± 0.13	2nd
Methods	79.61%	13.53 ± 0.99	3rd
Results	74.85%	8.23 ± 1.33	4th
Discussion	64.75%	2.59 ± 0.37	5th
Abstract	55.29%	6.64 ± 1.25	6th
Other	16.31%	0.98 ± 0.69	Lowest

### “LLM-as-a-Judge” performance

**Fig. 1:**
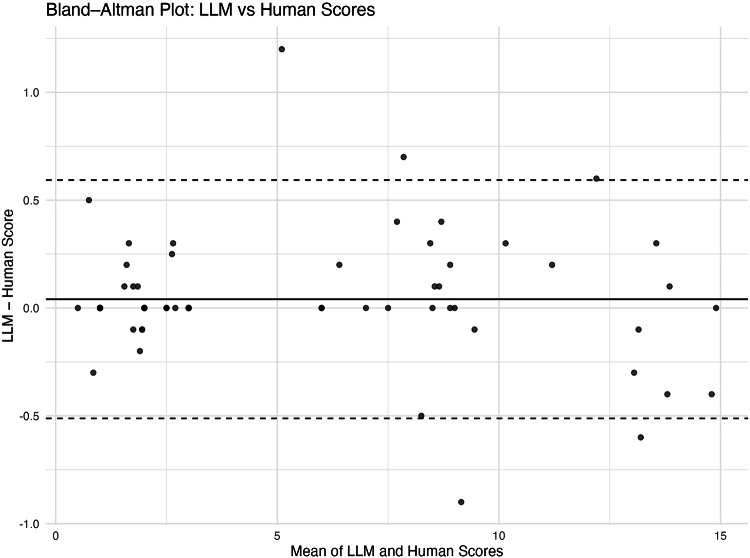
Bland-Altman Plot of LLM-Generated versus Human Scores.

To validate the “LLM-as-a-Judge” framework, we randomly selected five “synthetic” manuscripts from each topic for a total of ten manuscripts. These ten were evaluated separately by a human reviewer based on the PRISMA 2020 checklist. The agreement between LLM-generated scores and human scores was evaluated and shown with Bland–Altman analysis plots (Fig. 1), which showed the LLM behaves conservatively and consistently, as most of the points fell between the 95% Cl and differences are small and clustered near zero. Intraclass correlation coefficients (ICC) were stratified by manuscript section (Table 3). Almost all variability was due to manuscript difference instead of the measure method difference in the “Discussion” (ICC = 0.99, 95% CI: 0.97–1.00), “Methods” (ICC = 0.95, 95% CI: 0.81–0.99), “Other” (ICC = 0.97, 95% CI: 0.91–0.99), “Abstract” (ICC = 0.96, 95% CI: 0.84–0.99), and “Results” (ICC = 0.92, 95% CI: 0.72–0.98) sections. The “Introduction” section demonstrated that most of the variability was due to manuscript difference instead of the measure method difference (ICC = 0.87, 95% CI: 0.55–0.97), though with wider confidence intervals. For the “Title” section, the LLM consistently assigned a perfect score (1.0) across all manuscripts, precluding estimation of ICC due to zero variance in LLM scores.

**Table 3:** “LLM-as-a-judge” performance

Section	ICC	95% CI
Abstract	0.956	0.841–0.989
Discussion	0.992	0.969–0.998
Introduction	0.869	0.550–0.966
Methods	0.949	0.814–0.987
Other	0.974	0.906–0.993
Results	0.918	0.721–0.979
Title	All LLM scores = 1.0 (perfect); ICC not estimable	–

Intraclass correlation coefficients between LLM and human scores across manuscript sections. Ten manuscripts (five from each topic) were evaluated both by LLM and a human rater. The rating scores were compared with intraclass correlation coefficients.

*ICC* intraclass correlation coefficients, *Cl* confidence interval.

## Discussion

Meta-analyses and systematic reviews are powerful for evidence synthesis and are widely used in medical research^14^, especially when evidence is conflicting or unclear^15^. Their construction and writing can be very time-consuming, a process that takes weeks to months and that is vulnerable to inter‑reviewer variability and screening errors. We used motivating systematic reviews and meta-analyses studies from hepatology (carvedilol in compensated cirrhosis and anticoagulation in PVT) as ones to emulate using LLMs and found LLM with excellent performance in the article screening and manuscript rating stage, but suboptimal in the manuscript generating stage.

For the step of title and abstract screening, which is the most time-consuming aspect of performing meta-analyses and literature reviews^16^, our results suggested that LLM-based tools can substantially reduce the time required for manual review while maintaining acceptable accuracy. Our data demonstrated that LLMs achieved high sensitivity (82–93%) and specificity (95–99%) for identifying relevant studies, offering strong support for partial automation of the screening workflow. Both sensitivity and specificity surpassed prespecified thresholds of 80% in both clinical topics. These findings indicate that the LLM-based pipeline could reduce initial screening workloads, augmented by a more rapid human review of borderline or model-flagged cases, which helps catch residual errors.

The average time for manual screening of articles has ranged between 0.9–7 min per article^17^. This means that it may take up to 40–60 h for a study with 800 constituent articles. In our study, the carvedilol in compensated cirrhosis topic took 62 h of human reviewer time compared to 3 h and USD $82.50 via LLM-assisted screening. Similarly, the anticoagulation in PVT topic took 30 h of human reviewer time compared to 2 h and USD $38.40 by LLM-assisted screening. In both instances, LLM-assisted screening significantly reduced the human time required for screening at an acceptable cost.

Despite the LLM’s superior abilities in article screening and selection, there were substantial challenges for the loftier goal of automated drafting. In our analyses, we found that although the “Introduction” and “Methods” sections of the “synthetic” drafts received high PRISMA 2020 scores, the “Results” sections were prone to inaccuracies in extracting numerical data or generating spurious (e.g., “hallucinated”) findings. Consequently, the downstream “Discussion” sections often drew on these flawed outputs, further diminishing their reliability. These issues in data presentation highlight the risk of relying exclusively on machine-drafted sections without robust manual verification and editing.

One initial thought on the inaccuracies in extracting data from the studies included was the limited ability to process the tables and figures in the file. In response, we attempted another methodology to extract the relevant data by applying LangChain^18^, which is a software package that allows more effective parsing of data from unstructured tables and figures, as a pre-processing step. In this sensitivity analysis, we uploaded the pre-processed tables and figures as separate files and linked them to the original manuscript's text directions. Since there were difficulties in preserving the linkages between table columns, row labels, and figure captions, we also used LangChain functionalities to identify tables and figures and format them separately from the main body text to capture the data structure. However, performances did not improve, even when LangChain pre-processing was incorporated into the construction of RAG databases.

While there are very few precedents that exist for the end-to-end use of LLM-based systems to fully draft meta-analysis manuscripts, the inaccuracies and hallucinations we observed are consistent with well-documented limitations of LLMs in complex quantitative tasks. The capacity of LLMs to draft methodologically structured text has been reported in other studies, but their reliability for generating data-heavy outputs remains limited^19^.

In the current landscape of evidence synthesis, several platforms have now integrated AI to streamline the systematic review construction process. Tools such as Rayyan^20^ and HubMeta^21^ have long provided semi-automated screening based on active learning and keyword ranking, while SciSpace^22^ and Meta-MAR^23^ offer sophisticated PDF interaction and statistical automation. Our approach, however, diverges from these existing solutions in three critical ways. First, while commercial tools often operate as ‘black boxes’ with proprietary ranking algorithms, our study provides a transparent evaluation of LLM performance, achieving a high sensitivity (>90%) and specificity (>80%) that sets a clear benchmark for fully automated screening. Second, we identified a significant “performance ceiling” in data extraction and interpretation, a limitation often glossed over by commercial platforms but crucial for researchers to understand to avoid errors in meta-analysis. Finally, we also introduce an LLM-as-a-Judge framework grounded in PRISMA criteria for the automated grading of synthetic manuscripts. Unlike existing platforms that focus on the process of screening, our approach provides an automated evaluative layer, ensuring the final output adheres to international reporting standards. A recent 2024 review noted that most AI tools for systematic reviews lack transparency and explainability and are not formally validated against standard quality criteria like PRISMA^24^. This suggests that the future of LLM-assisted review lies not just in task automation but also in automated quality oversight.

Importantly, the LLM-assisted workflow presented here is not specific to hepatology. The prompting strategy, code structure, and methodological steps are domain independent and can be readily adapted to other clinical or scientific areas that require systematic literature review and meta-analysis. As such, the approach has broad potential applicability across disciplines, provided that domain-specific expertise is incorporated for topic selection, interpretation, and validation. Although our experiments were conducted on an institutional deployment of OpenAI models, the underlying methodology (few-shot prompting with explicit inclusion/exclusion criteria, RAG-based manuscript drafting, and LLM-as-a-Judge evaluation) is model-agnostic and could be implemented with other commercially available or open-source LLMs. We anticipate that performance characteristics may vary across models, and encourage future benchmarking studies to evaluate cross-platform reproducibility.

Several limitations of our study need to be acknowledged. First, despite large efficiency gains, screening was not fully automated: all model-flagged inclusions and ambiguous records still required human adjudication, and full-text eligibility decisions remained manual. Second, all experiments were run in the UCSF “Versa” platform, which is a secure, institution-hosted environment chosen for copyright and privacy considerations. We did not evaluate frontier public LLMs; therefore, performances with larger, non-distilled models may differ. Third, our evaluation exclusively focused on two hepatology topics (carvedilol in compensated cirrhosis and anticoagulation in PVT); generalizability to other topics and domains may be limited. Fourth, the “synthetic” automated drafting pipeline struggled with numerical extraction from tables/figures and produced spurious (“hallucinated”) results. The “synthetic” manuscripts, therefore, necessitated rigorous manual verification and were not immediately usable.

Despite these limitations, LLM-based workflow augmentation offers several potential benefits for clinicians, researchers, and policymakers in the medical field. Given the expanding literature, LLM-assisted screening may help keep pace with surging publication volumes. Furthermore, the capacity of these tools to draft structured sections can potentially free up expert time for higher-level synthesis and interpretation, rather than mechanical tasks. As our findings also emphasize, any draft text derived from an LLM must be validated to ensure accuracy and consistency.

Our pilot data suggests several directions for future development. First, refining data extraction pipelines, potentially via specialized modules that parse quantitative elements from figures or tables, may reduce hallucinations and enhance reproducibility. Second, improving prompt engineering techniques, including topic-specific phrases and definitions, might further boost screening performance and reduce false positives. Lastly, the design of integrated human-machine workflows should be explored, wherein domain experts vet “borderline” machine screening decisions and verify numeric data in the LLM-drafted results.

In summary, our findings suggest that an LLM-assisted approach to systematic reviews in hepatology can yield major efficiency gains in literature screening, with performance metrics that approach those of expert human screeners. LLM-assisted drafting of “synthetic” manuscripts, however, remains challenging due to data misrepresentation and hallucination errors. As LLMs continue to evolve, refining automated pipelines that reduce laborious tasks while preserving scientific rigor may help accelerate evidence synthesis across the continuum of hepatology and other medical field research.

## Methods

**Fig. 2:**
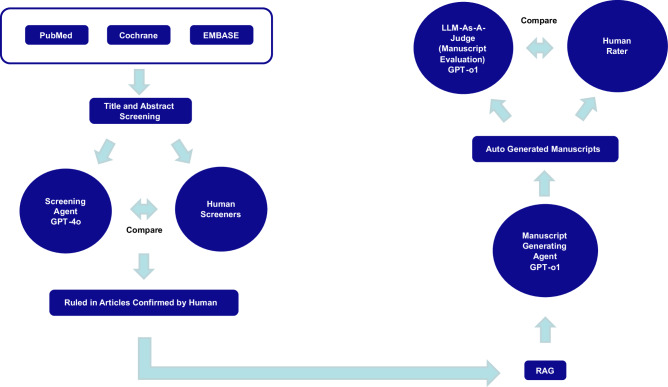
Study Workflow.

The whole workflow is shown in Fig. 2.

### Article filtering and screening

The first part of this study sought to test the abilities of LLMs for article search, screening, and filtering. We duplicated the inclusion and exclusion criteria for our motivating studies (Table 4). We conducted the article search with compatible search terms from PubMed, Cochrane, and EMBASE (see Supplementary Materials). We then utilized the University of California, San Francisco’s “Versa” platform, which is a Protected Health Information (PHI) compliant deployment of commercially available LLMs (specifically OpenAI’s GPT-4o), to filter and screen articles.

**Table 4:** Inclusion and exclusion criteria of the two motivating studies

Study	Inclusion criteria	Exclusion criteria
Carvedilol and cirrhosis	1. Clinical studies comparing carvedilol with no treatment, placebo, or esophageal variceal ligation for prevention of cirrhosis decompensation (including variceal bleeding) or for improving survival in compensated cirrhosis. 2. Study designs: clinical trials, case–control studies, or cohort studies.	1. Non-human studies. 2. Case reports, case series, reviews, meta-analyses, study protocols, and letters. 3. Article collections or conference proceedings (multiple abstracts in one source). 4. Non-English publications.
Anticoagulant therapy and PVT	1. Clinical studies comparing anticoagulant therapy versus no treatment in cirrhotic patients with portal vein thrombosis (PVT). 2. Outcomes of interest: variceal/non-variceal bleeding, PVT recanalization, and PVT progression. 3. Study designs: randomized clinical trials, cohort studies, and case–control studies.	1. Non-human (animal or in vitro) studies. 2. Case reports, case series, reviews, meta-analyses, study protocols, and letters to the editor. 3. Studies of PVT in non-cirrhotic patients. 4. Studies without an untreated (no anticoagulation) control group. 5. Studies not addressing anticoagulation effects on PVT in cirrhosis. 6. Studies of PVT arising after liver transplantation.

For each motivating study, we randomly selected 100 unique abstracts from articles initially identified via article search from PubMed, Cochrane, and EMBASE to develop the appropriate filtering prompts (Supplementary Material 1 and 2). Using human review as the gold standard, we iteratively refined the prompt until it achieved over 90% sensitivity for detecting relevant studies while maintaining specificity above 80% to minimize false positives. Through iterative adjustments, the prompt was optimized by providing curated examples (“few-shot” prompting), embedding explicit inclusion/exclusion criteria in concise bullet-point form, employing emphasis markers for critical elements, and streamlining language to minimize ambiguity. A detailed description of the prompt engineering strategy, including the iterative development process and an example, is provided in Supplementary Material 8.

Using the refined prompt, the LLM achieved sensitivity >90% and specificity >80% in the pilot set, demonstrating feasibility for broader validation (see Supplementary Material 3 and 4 for Final Prompts Used). We then used a held-out set of 100 randomly selected abstracts to validate the finalized prompt. The results of the screening were again compared to human review and calculated performance statistics, specifically sensitivity, specificity, negative predictive value, and positive predictive value.

Total human screening time includes the total amount of time human reviewers used to screen all the constituent articles. Total LLM-assisted screening time includes prompt generating, adjusting time, application programming interface code writing time and time used by the LLM to screen and process all the of articles.

### Construction of customized LLMs for meta-analyses drafting

After the initial article filtering process, we embedded the filtered articles into a customized LLM through Retrieval Augmented Generation (RAG)^25^. RAG is an enterprise architecture that supplements LLM abilities by providing external data to an information retrieval system. This augments and constrains the LLM output, meaning that a data set is vectorized and encoded using embedding models and then incorporated into the LLM by layering it on top of the LLM information retrieval and output processes. In the construction of the RAG databases, we directly uploaded the electronic text files, commonly in portable document format (.pdf), into the RAG system. This would give the LLM a compendium of files from which it was asked to construct a meta-analysis. In the prompts, the LLM were asked to provide their reasoning for which files were selected.

### “Synthetic” meta-analysis drafting

We use the term “synthetic” throughout this manuscript to describe manuscripts that were entirely machine-generated by the LLM pipeline without human authorship or editing; the term does not refer to fabricated or simulated data but rather to the automated, LLM-produced nature of the draft text and its embedded analyses. For the foundation model underlying the RAG infrastructure, we choose OpenAI-o1 Reasoning for drafting components of meta-analyses. We utilized the OpenAI-o1 Reasoning model instead of GPT-4o for this exercise because our pipeline requires reliable multi-step statistical reasoning (e.g., effect-size conversions, model-choice justification, and edge-case handling) and strict adherence to structured output schemas used by the RAG orchestrator. In our pilot runs, OpenAI-o1 produced fewer extraction and transformation errors, yielded more consistent JSON conforming to our schema, and was superior at self-checking intermediate calculations compared with fast, multimodal models like GPT-4o.

We constructed an RAG-LLM for each motivating example (e.g., “carvedilol in compensated cirrhosis” and “anticoagulation in PVT”). Each RAG-LLM was given a similar prompt to draft manuscripts (Supplementary Material 5). Within each active session, each RAG-LLM was prompted to generate the manuscript sections (e.g., Introduction, Methods, Results, Discussion, Abstract, and Title) continuously as sections were dependent on the previous section’s information. New sessions were created after the previous session completed its “manuscript,” to prevent cached memory from affecting the later outputs. In generating the prompts for each section, we provided the LLMs with: (1) general guidance on the type of information included for each manuscript section, (2) instructions to cite information from which they had used for statistical analyses, and (3) instructions to show any code or calculations for the compilation of actual meta-analyses. We did not provide any explicit guidance on which articles or abstracts to use from the pool of selected texts embedded in the RAG databases. Final prompts used to generate manuscript sections are featured in the Supplementary Material 5.

The LLM determines whether a quantitative meta-analysis is appropriate based on the included studies and extracted data, and, when applicable, selects the analytic model (e.g., fixed-effect or random-effects), summary measures, heterogeneity statistics, and study quality assessments dynamically rather than using methods predefined by the authors.

Using this methodology, we generated ten “synthetic” manuscripts for “carvedilol in compensated cirrhosis” and “anticoagulation in PVT.”

### Evaluation of LLM-generated “synthetic” meta-analyses

We utilized the “LLM-as-a-Judge” approach with the expanded PRISMA 2020 checklist to systematically assess the quality of each RAG-LLM-generated “synthetic” meta-analysis^26^. In our evaluation process, we utilized a separate instance of the OpenAI-o1 model as the “judge.” A new chat session was created to evaluate each section of the manuscript to prevent memory contamination and time-out errors due to excessive data and requests.

The “synthetic” manuscripts were divided into seven sections in the PRISMA 2020 checklist to be evaluated: Title, Abstract, Introduction, Methods, Results, Discussion, and Other. In the PRISMA 2020 checklist, each section had evaluation metrics that specified content to be included and quality of the content. For instance, for the “Objectives” metric in the “Introduction” section of the manuscript, the PRISMA 2020 checklist defines the evaluation item for this metric to be “*Provide an explicit statement of the objective(s) or question(s) the review addresses*,” and elements to be “*Provide an explicit statement of all objective(s) or question(s) the review addresses, expressed in terms of a relevant question formulation framework*” and “*If the purpose is to evaluate the effects of interventions, use the Population, Intervention, Comparator, Outcome (PICO) framework or one of its variants, to state the comparisons that will be made*”^11^.

Using the PRISMA 2020 checklist metrics (Supplementary Material 6), items, and elements, we generated specific prompts for each evaluation metric and asked the LLM “judge” to grade the appropriate section of the “synthetic” manuscript. Each checklist metric was assigned a continuous score by the LLM “judge” from 0–1 with 0 indicating non-compliance and 1 indicating compliance. The LLM “judge” was also asked to provide a brief rationale for assigning the score it did per checklist item as well as a citation from the manuscript’s section justifying its rationale. We defined the sum of all the sections evaluated as the manuscript’s composition score, with the maximum score being 53 points (see Supplemental Material 7).

To validate the performance of “LLM-as-a-judge,” we selected a total of ten “synthetic” manuscripts (five from the “carvedilol in compensated cirrhosis” and five from “anticoagulation in PVT”) and compared the “LLM” judge’s performance to “gold-standard” human evaluations via Bland-Altman analysis. The mean difference (bias) and 95% limits of agreement were calculated, and results were visualized using Bland-Altman plots. We also calculated intraclass correlation coefficients (ICC) to show the variability differentiation.

## Supplementary information

Supplementary Information

## Data Availability

All data used in this study were derived from previously published articles available in the scientific literature.

## Code availability

Analysis code is publicly available at https://github.com/YZ-334455/PCORI_Meta_LLM.git.
