# Supplementary material for: Closing the screening gap but not the writing gap: a two-topic evaluation of LLMs for systematic reviews and meta-analyses in hepatology
Source: NPJ Gut Liver. 2026 Jun 22;3(1):21. doi: 10.1038/s44355-026-00068-w (PMC13287001; doi:10.1038/s44355-026-00068-w)
Supplement: Supplementary file 1 — Supplementary Information [file 44355_2026_68_MOESM1_ESM.pdf]

Supplemental Materials 1: ***Article Search Query for Carvedilol in Compensated Cirrhosis***

Pubmed

((carvedilol [Title/Abstract]) OR (Dilatrend [Title/Abstract]) OR (Querto [Title/Abstract]) OR (Coreg [Title/Abstract]) OR (Coropres [Title/Abstract]) OR (Eucardic [Title/Abstract]) OR (Kredex [Title/Abstract]) OR (BM 14190 [Title/Abstract])) AND ((Cirrhosis [Title/Abstract])OR (portal hypertension [Title/Abstract]) OR (liver [Title/Abstract]) OR (Variceal bleeding [Title/Abstract]) OR (variceal hemorrhage [Title/Abstract]) OR (Esophageal [Title/Abstract]) OR (ascites [Title/Abstract]) OR (hepatorenal syndrome [Title/Abstract]) OR (encephalopathy [Title/Abstract]))

Embase

('carvedilol':ab,ti OR 'Dilatrend':ab,ti OR 'Querto':ab,ti OR 'Coreg':ab,ti OR 'Coropres':ab,ti OR 'Eucardic':ab,ti OR 'Kredex':ab,ti OR 'BM 14190':ab,ti) AND ('Cirrhosis':ab,ti OR 'portal hypertension':ab,ti OR 'liver':ab,ti OR 'Variceal bleeding':ab,ti OR 'variceal hemorrhage':ab,ti OR 'Esophageal':ab,ti OR 'ascites':ab,ti OR 'hepatorenal syndrome':ab,ti OR 'encephalopathy':ab,ti)

Cochrane

("BM 14190":ti,ab,kw OR "Kredex":ti,ab,kw OR "Eucardic":ti,ab,kw OR "Coropres":ti,ab,kw OR "Coreg":ti,ab,kw OR "carvedilol":ti,ab,kw OR "Dilatrend":ti,ab,kw OR "Querto":ti,ab,kw) AND ("cirrhosis":ti,ab,kw OR "portal hypertension":ti,ab,kw OR "liver":ti,ab,kw OR "variceal bleeding":ti,ab,kw OR "IBD":ti,ab,kw OR "esophageal":ti,ab,kw OR "ascites":ti,ab,kw OR "hepatorenal syndrome":ti,ab,kw OR "encephalopathy":ti,ab,kw)

Supplemental Materials 2: ***Article Search Query for Anticoagulation in PVT***

PubMed

((“portal vein”[MeSH Terms] OR (“portal”[All Fields] AND “vein”[All Fields]) OR “portal vein”[All Fields]) AND (“thrombosis”[MeSH Terms] OR “thrombosis”[All Fields])) AND (“liver cirrhosis”[MeSH Terms] OR (“liver”[All Fields] AND “cirrhosis”[All Fields]) OR “liver cirrhosis”[All Fields] OR “cirrhosis”[All Fields] OR “fibrosis”[MeSH Terms] OR “fibrosis”[All Fields]) AND (“anticoagulants”[Pharmacological Action] OR “anticoagulants”[MeSH Terms] OR “anticoagulants”[All Fields] OR “anticoagulant”[All Fields]) AND “humans”[MeSH Terms]

ISI Web of Science

ALL = ("portal" AND "vein")
AND ALL =("thrombosis")
AND ALL =("cirrhosis" OR "fibrosis")
AND ALL =("anticoagulants" OR "anticoagulant")
AND ALL =("human" OR "humans")

the Cochrane database

("portal":ti,ab,kw AND "vein":ti,ab,kw)
AND ("thrombosis":ti,ab,kw)
AND ("cirrhosis":ti,ab,kw OR "fibrosis":ti,ab,kw)
AND ("anticoagulants":ti,ab,kw OR "anticoagulant":ti,ab,kw)
AND ("human":ti,ab,kw OR "humans":ti,ab,kw)

Supplemental Materials 3: *Screening Prompt for Carvedilol in Compensated Cirrhosis*

I am screening articles for a meta-analysis systemic review. This is the first step to only screen for title and abstract to decide if they need go forward for full text screening or can be ruled out only based on title and abstracts. The rule-in and rule-out criteria are as below. The articles need to meet all rule-in criteria and none of the rule-out criteria to be selected to go for full text screening.

Rule-in Criteria:

1. Studies need to compare carvedilol with no treatment, placebo, or esophageal variceal ligation on the effect of preventing decompensation of cirrhosis (including variceal bleeding) or improving survival in patients with compensated cirrhosis. **Any mention of variceal bleeding in the result, even if no bleeding occurred, should be considered relevant for inclusion. Both primary and secondary outcomes related to decompensation or mortality should be considered.**
2. Studies need to be clinical trials, case control studies or cohort studies.

Rule-out criteria:

1. Studies that are not performed on human being patients are excluded.
2. Case reports, case series, reviews, meta-analysis, study protocol and letters are excluded.
3. If the article has multiple studies or abstracts, such as article collections or conference books, it need to be excluded.
4. Studies not written in English are excluded.

Study needs to contain following analysis

- Population: patients with compensated cirrhosis
- Treatment group: receiving carvedilol
- Control group: either no treatment, placebo or only with esophageal variceal ligation(the comparation can be in subgroups; but ok to have other groups; and as long as the there are group or subgroup of carvedilol and groups or subgroup of no treatment or only with esophageal variceal ligation)
- Outcome: decompensation of cirrhosis rate(including variceal bleeding) or mortality(survival rate). Both of them can be primary or other outcomes (Ok to evaluate other effects, as long as the decompensation or mortality are evaluated/reported).

Very important point

1. **Clarify the Importance of Outcomes other than primary outcomes**: Emphasize that both primary and other outcomes related to decompensation of cirrhosis or mortality should be considered for inclusion.
2. **Highlight Reporting of Variceal Bleeding**: Extremely pay attension to that any mention of bleeding, regardless of whether variceal bleeding occurred, is sufficient for inclusion.
3. **Explicitly Include Studies with No Bleeding Events**: Add a specific note that studies reporting no bleeding events should still be considered if they mention variceal bleeding as a result.

Clarifications：

1. 1.** If the treatment group has carvedilol and other β blockers, and there is a subgroup analysis where carvedilol is compared to placebo or no treatment, the article should be ruled in for full-text screening **.
2. 2.** It is acceptable for our target population and comparison to occur only in a subgroup of the study, if the subgroup analysis meets the rule-in criteria.**
3. Decompensation of cirrhosis is defined as cirrhosis with at least one of the following complications: Variceal hemorrhage, Ascites, Spontaneous bacterial peritonitis, Hepatic encephalopathy, Hepatorenal syndrome, Hepatopulmonary syndrome, Hepatic hydrothorax,

Portopulmonary hypertension. So reoorts of any of above should be considered as evaluation of decompensation of cirrhosis.

4. Hepatocellular carcinoma is NOT included as one of the criteria of decompensation of cirrhosis, due to its development is not only due to cirrhosis. But if the population included before therapy only have patients with hepatocellular carcinoma, this article should be excluded.
5. If the treatment group has carvedilol and other β blockers and it is unclear if there is a subgroup of carvedilol analyzed per the title and abstract, the article should be kept ruled in for next step, full-text screening.
6. If it only mentioned patients with cirrhosis, but cannot find if there is compensated cirrhosis subgroup, the article should not be ruled out due to the unclear if there is compensated cirrhosis subgroup. And if not meeting other rule-out criteria and meeting, it should be ruled-in for next step, full-text screening.
7. It is ok our target population and comparation only happening in the subgroup of the study.
8. If the carvedilol is both in treatment and control group, the study should be excluded.
9. Preventing cirrhosis decompensation includes primary prophylaxis of variceal bleeding--prevent first-time variceal bleeding. **If the study reports on variceal bleeding result, regardless of whether bleeding occurred, it should be considered for full text screening, regardless of whether it is the primary or other outcome.**
10. Child-Pugh class A and B cannot tell if compensated or decompensated. Child-Pugh class C is surely decompensated.

Here are some examples of articles ruled out or ruled in.

1. <u>Effects of carvedilol for heart failure in patients with functionally univentricular heart</u>
   Objective: Our purpose was to evaluate the clinical effects of Carvedilol for heart failure in patients with functionally univentricular heart. Methods: Between 2002 and 2008, we used Carvedilol in treating patients with functionally univentricular heart exhibiting heart failure (n = 51). We classified them into three groups according to the status of each patient including status post Fontan operation(P), status post bidirectional Glenn (G), and those undergoing neither Glenn nor Fontan (NF). We compared clinical parameters before and after implementing Carvedilol therapy. The parameters included cardiothoratic ratio (CTR), ventricular end-diastolic pressure (EDP), central venous pressure (CVP), ventricular ejection fraction (EF), serum brain natriuretic peptide level (BNP), and dosages of diuretics. We also investigated the New York Heart Association (NYHA) classification, degree of artrioventricular valvular regurgitation (AVVR), and presence of arrhythmia. Results: Carvedilol therapy was started at the mean age of 10.1 y (range, 1m to 34.8 y). The initial dose was 0.04 (range, 0.01 to 0.18)mg/kg/day and the maximum dose was 0.42 (range, 0.01 to 0.92)mg/kg/day. The main reason for starting Carvedilol was heart failure associated with ventricular contractile dysfunction (n = 35), pleural effusion (n = 11), ascites (n = 1), arrhythmia (n = 10), AVVR (n = 11), protein-losing enteropathy (n = 1) and frequent hospitalization (n = 3). The patients were on diuretics (furosemide, hydrochlorothiazide or spironolactone; n = 43), enalapril (n = 34), pimopendan (n = 9), digoxin (n = 19) and losartan potassium (n = 1). The CTR. improved significantly from 59 to 46% (p<0.01), and the dosage of diuretic reduced significandy (p<0.01). The EP also significant improved in all patients (from 35 to 40%;p < 0.05). This improvement was especially prominent in the Fontan group (from 35 to 45%; p<0.05). Clinical signs, symptoms and NYHA classes were also improved. Conclusions: We think that Carvedilol might play an important role in treating heart failure associated with functionally univentricular heart.
   This should be Ruled-out. Because it only evaluated effect on heart failure instead of preventing decompensation of cirrhosis or improving survival.

2. <u>To compare endoscopic variceal ligation + carvedilol versus endoscopic variceal ligation + propranolol on hepatic vein pressure gradient reduction at 1 month in patients with first episode of esophageal varix bleed: open label randomized trial</u>
   Aim: Primary objective of this study was to compare endoscopic variceal ligation (EVL) plus Propranolol versus EVL plus Carvedilol on reduction of HVPG after one month of therapy with secondary objective of comparison of rate of rebleeding after index esophageal variceal bleed in Child A/B cirrhosis. Methods: Patients of Child A/B cirrhosis presenting to emergency, from June 2014 to December 2013, with index esophageal variceal bleed received standard treatment (somatostatin therapy followed by EVL) following which HVPG was measured and patients were randomized to Propranolol or Carvedilol group if HVPG was >12 mm. Propranolol and Carvedilol were increased gradually with target heart rate of 55-60 beats per minute with maximum tolerable dose. HVPG was again measured at 1 month of treatment. Patients were followed up till 1 year to compare rates of rebleeding. Results: Of 129 patients of index esophageal variceal bleed, 59 patients were randomized into Carvedilol (n = 30) and Propranolol (n = 29). At 1 month of treatment, decrease in heart rate, mean arterial blood pressure (MAP) and HVPG was significant within each group (P = .001). Number of HVPG responders (HVPG decrease >20% or below 12 mm Hg) was significantly more in Carvedilol group (22/29) as compared to Propranolol group (14/28), P-value = .04. There was only 1 rebleed in each group at 1 month. At 1 year, rebleed occurred in 4/21 in Carvedilol group out of which two was in responders (2/17) and two in non-responders (2/4). Rebleed at 1 year, in Propranolol group occurred in eight patients (8/ 17) out of which three was in responders (3/10) and five in non-responders (5/7). Attrition was one patient in first month in each group, eight at 1 year in Carvedilol group and eleven in Propranolol group. Conclusion: Carvedilol is more effective in reducing portal pressure in patients with cirrhosis than Propranolol. Though a larger study is required to substantiate this, but results in this study are promising for Carvedilol.
   This should be Ruled-out. Because it only had decompensation patients and the control group had other treatment other than EVL
3. <u>Clinical algorithms for the prevention of variceal bleeding and rebleeding in patients with liver cirrhosis</u>
   Portal hypertension (PH), a common complication of liver cirrhosis, results in development of esophageal varices. When esophageal varices rupture, they cause significant upper gastrointestinal bleeding with mortality rates up to 20% despite state-of-the-art treatment. Thus, prophylactic measures are of utmost importance to improve outcomes of patients with PH. Several high-quality studies have demonstrated that non-selective beta blockers (NSBBs) or endoscopic band ligation (EBL) are effective for primary prophylaxis of variceal bleeding. In secondary prophylaxis, a combination of NSBB + EBL should be routinely used. Once esophageal varices develop and variceal bleeding occurs, standardized treatment algorithms should be followed to minimize bleeding-associated mortality. Special attention should be paid to avoidance of overtransfusion, early initiation of vasoconstrictive therapy, prophylactic antibiotics and early endoscopic therapy. Pre-emptive transjugular intrahepatic portosystemic shunt should be used in all Child C10-C13 patients experiencing variceal bleeding, and potentially in Child B patients with active bleeding at endoscopy. The use of carvedilol, safety of NSBBs in advanced cirrhosis (i.e. with refractory ascites) and assessment of hepatic venous pressure gradient response to NSBB is discussed. In the present review, we give an overview on the rationale behind the latest guidelines and summarize key papers that have led to significant advances in the field.
   This should be Ruled-out. Because it is a review.

4. <u>Effects of carvedilol on the expression of tlr4 and its downstream signaling pathway in the liver tissues of rats with cholestatic liver fibrosis</u>
Objectives: This study was designed to investigate the effects of carvedilol on the expression of TLR4 and its downstream signaling pathway in the liver tissues of rats with cholestatic liver fibrosis and provide experimental evidence for clinical treatment of liver fibrosis with carvedilol. Methods: A total of fifty male Sprague Dawley rats were randomly divided into five groups (10 rats per group): sham operation (SHAM) control group, bile duct ligation (BDL) model group, low-dose carvedilol treatment group (0.1mg·kg-1·d-1), medium-dose carvedilol treatment group (1mg·kg-1·d-1), and high-dose carvedilol treatment group (10mg·kg-1·d-1). Rat hepatic fibrosis model was established by applying BDL. Forty-eight hours after the operation, carvedilol was administered twice a day. The blood and liver were simultaneously collected under the aseptic condition for further detection in two weeks after the operation. The alanine aminotransferase (ALT), aspartate aminotransferase (AST), total bilirubin (TBil) and albumin (Alb) in serum were measured. HE and Masson staining were used to determine hepatic fibrosis degree. Hydroxyproline assay was employed to detect liver collagen synthesis. Western Blot was used to measure the expression of TLR4, NF-κB p65 and β-arrestin2 protein. Quantitative analysis of TLR4, MyD88, TNF-α and IL-6 mRNA was performed by Realtime-PCR. Results: Compared with the SHAM group, the BDL group showed obvious liver injury, increased levels of inflammatory factors, and continued progression of liver fibrosis. The above changes in the BDL group were alleviated in the carvedilol treatment groups. The improvement effects augmented as dosages increased. In addition, compared with the BDL group, the reduction of the expressions of TLR4, MyD88 and NF-κB p65 in liver tissues and the increase of the expression of β-arrestin2 in the high-dose carvedilol group were more significant. Conclusion: Carvedilol can reduce the release of inflammatory mediators by down-regulating TLR4 expression and inhibiting its downstream signaling pathway, thus playing a potential therapeutic role in cholestatic liver fibrosis.
This should be Ruled-out. Because it is a study performed on animals not on human being patients.
5. <u>Carvedilol vs. esophageal variceal band ligation in the primary prophylaxis of variceal hemorrhage: A multicentre randomized controlled trial</u>
Background & Aims Esophageal variceal bleed is a major problem in patients with cirrhosis. Endoscopic variceal ligation (EVL) has been shown to be equal to or better than propranolol in preventing first bleed. Carvedilol is a non-selective β blocker with alpha-1 adrenergic blocker activity. Hemodynamic studies have shown carvedilol to be more effective than propranolol at reducing portal pressure. We compared efficacy of carvedilol with EVL for primary prophylaxis of esophageal variceal bleed. Methods Cirrhotic patients with esophageal varices were randomized to carvedilol 12.5 mg daily or EVL at three university hospitals of Pakistan. End points were esophageal variceal bleeding, death or liver transplant. Results Two hundred and nine patients were evaluated. Eighty two and eighty six patients were randomized in carvedilol and EVL arms respectively. Mean age was 48 ± 12.2 years; 122 (72.7%) were males; 89.9% had viral cirrhosis; mean Child-Pugh score was 7.3 ± 1.6 and mean follow up was 13.3 ± 12.1 months (range 1-50 months). Both EVL and carvedilol groups had comparable variceal bleeding rates (8.5% vs. 6.9%), bleed related mortality (4.6% vs. 4.9%) and overall mortality (12.8% vs. 19.5%) respectively. Adverse events in carvedilol group were hypotension (n = 2), requiring cessation of therapy, while transient nausea (n = 18) and dyspnea (n = 30) resolved spontaneously. In the EVL arm, post banding ulcer bleed (n = 1) and chest pain (n = 17), were termed as serious adverse events while transient dysphagia (n = 58) resolved without treatment. Conclusions Although our study is underpowered, the findings suggest that carvedilol is probably not superior to EVL in preventing first variceal bleed in patients with viral cirrhosis.

This is a study that should be ruled in to go for full text screening. Because this is a clinical trial comparing carvedilol with Endoscopic variceal ligation on the effect of preventing decompensation--variceal bleeding and effect of mortality on cirrhotic patients. Although if all cirrhotic patients are compensated or there is a separate compensated group, then it should be checked in full text screening.

6. <u>A prospective, double-blind, randomized placebo-controlled trial of carvedilol for early primary prophylaxis of esophageal varices in cirrhosis</u>
   Background and Aims: Gastroesophageal variceal (GEV) hemorrhage is a major complication of portal hypertension resulting from cirrhosis of the liver. The risk of bleeding from small (≤5 mm) varices is 7 % at 2 years. Median rate of progression to larger varices is 5% to 23% per year. Carvedilol, a non-cardio selective vasodilating beta-blocker, has been claimed to be as effective in preventing variceal bleeding as EVL. We compared the efficacy and safety of carvedilol against placebo in the prevention of growth of small to large varices. Methods : One hundred and seventy-five consecutive patients with cirrhosis and portal hypertension with small esophageal varices (<5 mm in size and no red color signs) were prospectively enrolled from November 2010 to December 2012. Eighty-eight patients received carvedilol (Group A) and 87 patients received matched placebo (Group B). Upper gastrointestinal endoscopy was done at baseline, 6 months and 12 months. Fibroscan and HVPG were done at baseline and at 1 year of follow up. The primary endpoint of the study was prevention of development of large (>5 mm) varices. The secondary endpoints were adverse events and mortality in both groups. Results : Sixty-three patients in Group A and 69 patients in Group B completed 1 year follow up. The mean CTP and MELD in the carvedilol arm was 7.31±2.1 and 13.65±5.4 while in the placebo arm was 7.48±2.2 and 14.2±5.28 respectively. The mean carvedilol dose was 11.923±2.05 mg/day and target heart rate achieved in Group A was 58±3 beats per minute. Baseline HVPG in Group A and B were 15.3±3.9 and 16.06 ±5.7 respectively. After 1 year of treatment, there was no statistical difference in reduction of HVPG in the two groups (p-0.224). There was no correlation between change in HVPG and fibro scan in carvedilol and placebo group after the period of 1 year (p=0.415 vs. p=0.799). Twelve (19 %) patients in Group A and 14 (20.1 %) in Group B developed large esophageal varices (p=ns). The actuarial probability of prevention of development of large esophageal varices at 30 month was also not different between the groups (p-0.218). The kappa index for assessment of variceal size was <5 %. There were no difference in adverse events reported (7.9% in GroupAvs. 1.4% in Group B, p=0.109). No patient bled from varices in either group. Conclusion: This is the first RCT (NCT01196507) showing that while carvedilol was well tolerated, it was not effective in preventing growth of small to large esophageal varices. The drug was also not effective in achieving significant reduction in HVPG in this group of patients at 12 months.

   This is a study that should be ruled in to go for full text screening. Because this is a clinical trial comparing carvedilol with placebo on several outcomes. It evaluated mortality as secondary outcome and decompensation (variceal bleeding). No patient bled from varices in either group. Although primary outcome and several other outcomes are not relevant, as long as one of our target outcomes, decompensation (including variceal bleeding) and mortality (survival rate), are evaluated, the article should be ruled in for the full text screening.

I want to check the following article, with only title and abstract. It should be ruled in for full text screening or ruled out based on the title and abstract. If the study is ruled out, the ruled-out reason should be listed.

The corresponding output would be strictly in following two separated parts:

- First line should on have "0" or "1": 0 =rule out; 1=rule in for full text screening.
- Second line: "Reason of the decision of ruling in or ruling out"

Supplemental Materials 4: *Screening Prompt for Anticoagulation in PVT*

I am screening articles for a meta-analysis systemic review. This is the first step to only screen for title and abstract to decide if they need go forward for full text screening or can be ruled out only based on title and abstracts. The rule-in and rule-out criteria are as below. The articles need to meet all rule-in criteria and none of the rule-out criteria to be selected to go for full text screening.

We will assess the impact of anticoagulant therapy versus no treatment on portal vein thrombosis (PVT) recanalization and progression in patients with cirrhosis. Outcomes of interest include, but are not limited to, variceal and non-variceal bleeding, PVT recanalization, and PVT progression.

Inclusion (Rule-in) Criteria:

1. Clinical studies comparing anticoagulant therapy versus no treatment in cirrhotic patients with PVT.
2. Study designs: randomized clinical trials, cohort studies, and case–control studies.

Exclusion (Rule-out) Criteria

1. Non-human (animal or in vitro) studies.
2. Case reports, case series, reviews, meta-analyses, study protocols, and letters to the editor.
3. Studies of PVT arising in non-cirrhotic patients.
4. Studies without an untreated (no anticoagulation) control group.
5. Studies not addressing anticoagulation effects on PVT in cirrhosis.
6. Studies of PVT that developed after liver transplantation.

Study needs to perform following analysis

- Population: patients with cirrhosis and PVT
- Treatment group: receiving anticoagulant therapy
- Control group: no anticoagulant therapy
- Outcome: include, but are not limited to, variceal and non-variceal bleeding, PVT recanalization, and PVT progression.

######

Here are some examples of articles ruled out or ruled in.

1. <u>Successful use of danaparoid in the treatment of portal vein thrombosis that developed in a warfarin-administered hepatitis C virus-related cirrhosis patient</u>
   An 84-year-old woman with hepatitis C virus-related cirrhosis, hepatocellular carcinoma and atrial fibrillation, who presented with hematemesis, was initially treated with endoscopic variceal ligation (EVL) for an esophageal varix hemorrhage. However, computed tomography (CT) upon admission had revealed portal vein thrombosis, despite having received warfarin for existing atrial fibrillation. We subsequently initiated a 2-week treatment with danaparoid; warfarin being discontinued to reduce the risk of re-hemorrhage. A follow-up CT after treatment revealed complete reduction of the portal vein thrombosis. This is the first successful report of danaparoid use in the treatment of portal vein thrombosis that developed in a patient who had received warfarin.
   This should be Ruled-out. Because this is a case report.
2. <u>Effectiveness of edoxaban in portal vein thrombosis associated with liver cirrhosis.</u>
   Portal vein thrombosis (PVT) worsens the long-term prognosis of patients with cirrhosis; however, the optimal treatment remains to be determined. Reports on the efficacy of direct oral anticoagulants are increasing, and further evidence is needed. Therefore, we investigated the effectiveness of treatment with edoxaban in patients with PVT. We retrospectively reviewed the outcomes of edoxaban and warfarin as antithrombotic therapies for PVT. The median overall survival time was 4.2 years in patients with PVT, with a 1-year survival rate of 70.7% and a 5-year survival rate of 47.9%. The leading cause of death was hepatocellular carcinoma. The overall

response rate for thrombolysis in the edoxaban group was 76.7% compared to 29.4% in the warfarin group, and edoxaban significantly improved PVT compared to warfarin. In addition, edoxaban provided long-term improvement of PVT. Warfarin, on the other hand, was temporarily effective but did not provide long-term benefits. The Child-Pugh and albumin-bilirubin scores did not change after edoxaban or warfarin use. No deaths occurred due to adverse events associated with edoxaban or warfarin. Edoxaban as a single agent can achieve long-term recanalization without compromising the hepatic reserves. Edoxaban is easy to initiate, even in an outpatient setting, and could become a major therapeutic agent for the treatment of PVT.

This should be Ruled-out. Because it has no control group of no anticoagulation therapy. Both groups were no anticoagulation therapy, warfarin and edoxaban.

3. <u>Prognostic factors in noncirrhotic patients with splanchnic vein thromboses.</u>
"Splanchnic vein thrombosis (SVT), not associated with cancer or liver cirrhosis, is a rare event and scanty data are available on its natural history, long-term prognosis, and treatment. In this study 121 SVT patients consecutively seen from January 1998 to December 2005 were included and 95 of them were followed up for a median time of 41 months. Screening for thrombophilic factors was performed in 104 patients. New thrombotic or bleeding episodes were registered and anticoagulant therapy was performed according to pre-established criteria._SVT was an incidental finding in 34 (28.1%) patients; 34 (28.1%) presented with abdominal infarction; 39 (32.2%) had bowel ischemia or acute portal vein thrombosis; 14 (11.6%) had bleeding from portal hypertensive sources. Survival rates at 1, 3, and 7 yr were 95%, 93.3%, and 89.6%, respectively; 87.5% of deaths occurred at onset of SVT as complications of intestinal infarction. Patients with isolated portal vein thromboses had symptoms and intestinal infarction in 16/41 (39%) and 0/41 (0%) of the cases, respectively, whereas superior mesenteric vein thromboses, isolated or not, were associated with symptoms and intestinal infarction in 69/75 (92%) and 34/75 (45%), respectively. During the follow-up 14 (14.7%) suffered from 39 episodes of gastrointestinal bleeding with no deaths. A previous gastrointestinal bleed was associated with new hemorrhagic events during follow-up. New venous thrombotic episodes occurred in 10 of 95 patients (10.5%), of which 73% were in the splanchnic area. Seven out of these 10 patients had a chronic myeloproliferative disease (MPD) and none was on anticoagulation._Anticoagulant therapy was effective to obtain recanalization of acute SVT in 45.4% of patients and preserved patients from recurrent thrombosis when given lifelong."
This should be Ruled-out. Because it is not about cirrhosis.
4. <u>Anticoagulation in Cirrhosis and Portal Vein Thrombosis Is Safe and Improves Prognosis in Advanced Cirrhosis.</u>
The role of portal vein thrombosis (PVT) in the natural history of cirrhosis is controversial._We analyzed the safety and effect of anticoagulant therapy (AT) on PVT recanalization and orthotopic liver transplant (OLT)-free survival._Eighty consecutive patients from a prospective registry of cirrhosis and non-tumoral PVT at a tertiary center were analyzed. AT effect on PVT recanalization and OLT-free survival was determined by time-dependent Cox regression analysis. Average MELD score was 15 ± 7. Portal hypertension-related complications at PVT diagnosis were present in 65 (81.3%) patients. Isolated portal vein trunk/branch thrombosis was present in 53 (66.3%) patients. AT was started in 37 patients. AT was stopped in 17 (45.9%) patients, in 4 (10.8%) due to bleeding events. No variceal bleeding occurred while on AT. Anticoagulation was restarted in 6/17 (35.2%) patients due to rethrombosis. In 67 patients with adequate follow-up imaging, AT significantly increased the rate of PVT recanalization compared with those who did not receive anticoagulation [51.4% (18/35) vs 6/32 (18.8%), p = 0.005]. OLT-free survival after a median follow-up of 25 (1-146) months was 32 (40%). Although there was no significant effect of AT on overall OLT-free survival, OLT-free survival was higher among patients with MELD ≥ 15

receiving AT compared to those who did not (p = 0.011). Baseline MELD at PVT detection independently predicted PVT recanalization (HR 1.11, 95% CI 1.01-1.21, p = 0.027) and mortality/OLT (HR 1.12, 95% CI 1.05-1.19, p < 0.001)._Although AT did not improve overall OLT-free survival, it was associated with higher survival in advanced cirrhosis. Anticoagulation increased PVT recanalization and should be maintained after PVT recanalization to avoid rethrombosis.

This should be Ruled-in. Because it is a study analyzing the effect of anticoagulant therapy on patient with cirrhosis and PVT. Safety and effect of anticoagulant therapy on PVT recanalization and orthotopic liver transplant-free survival were analyzed. Patients on anticoagulant therapy were compared with patients not on anticoagulant therapy.

######

I want to check the following article, with only title and abstract. It should be ruled in for full text screening or ruled out based on the title and abstract. If the study is ruled out, the ruled-out reason should be listed.

The corresponding output would be strictly in following two separated parts:

- First line should on have "0" or "1": 0 =rule out; 1=rule in for full text screening.
- Second line: "Reason of the decision of ruling in or ruling out"

Supplemental Materials 5: ***Manuscript Generation Prompt for Carvedilol in Compensated Cirrhosis and Anticoagulation in PVT***

Write a title, abstract, introduction, methods, results, discussion, and other section for a meta-analysis based on the uploaded files. The title should capture the topic and goal of the meta-analysis. The abstract should outline the purpose, important data, statistical analyses and methods, and summarize the results/conclusion of the meta-analysis. The abstract should include a brief summary of the methods (e.g. sources, eligibility, RoB), results (number of studies, effect of each study), interpretation and certainty of the meta-analysis, and the registration of the meta-analysis.

The introduction should include background information and context from each of the included studies, as well as detail the primary purpose behind the meta-analysis.

The methods section should describe the data from each of the studies and explain the analyses used in each of the individual studies, as well as the statistical analyses used in the meta-analysis. It should include protocol access, the support/funder role, and competing interests of each study. It should have the eligibility criteria and exclusion criteria for each of the studies. It should describe how the data were collected for each study and include the outcomes and variables of each study. The methods section should include risk-of-bias assessment and any effect measures. It should include synthesis criteria to decide study grouping. There should be heterogeneity metrics and heterogeneity analysis and exploration (e.g. subgroup, meta-regression, etc.). There should also be sensitivity analyses, certainty assessment methods (e.g. GRADE), and reporting-bias assessments. The methods section should provide definitions of terms and statistical methodologies, as well as why they were selected and how they are appropriate for this meta-analysis. The methods section should include all R code used for calculations, performance of statistical analyses, and plots/figures.

The results section should discuss the final analysis and interpret the data in a statistical and clinical context. It should go over significant findings and explain what they mean, as well as for plausible reasons explaining the significance. The results section should include the study selection, showing a flow diagram with numbers. There should be a study characteristic table and risk-of-bias per study. It should discuss the results of the individual studies and syntheses (pooled estimates). The results section should interpret and explain the heterogeneity statistics, sensitivity analyses results, reporting-bias assessments, and certainty of evidence.

In the discussion section, compare the results of this meta-analysis with the individual studies as well as existing literature on the topic of liver disease. The discussion section should include cross-study comparisons and discuss whether the findings of the meta-analysis align or not align with individual studies and explain potential reasons why. The discussion section should also explain the potential impact of this meta-analysis on the field. It should discuss the implications of this meta-analysis manuscript on current practice, policy, and research. It should also include general conclusions linked to objectives.

The other section should discuss limitations of the meta-analysis and how each limitation and/or bias was addressed or handled. If it was not handled, explain the reason why it was not or was unable to be addressed. Explore the limitations of the individual studies used in the meta-analysis as well. It should also include limitations of evidence and review processes of each individual study and the meta-analysis manuscript. The other section should also include whether the data, code, and/or materials used in each study were publicly available or not. If it was not publicly available, it should explain how to get access or explain why it is not publicly available. The other section should also include the location of the materials (either the URL or DOI).

Cite the studies included in the meta-analysis and properly extract data without hallucinating numbers. Write everything in complete sentences.

Supplemental Materials 6: *PRISMA 2020 Expanded Checklist (with Abstract Sub-items)*

Section & Topic	Item #	Checklist Item
TITLE	1	Identify the report as a systematic review.
ABSTRACT	2a	Identify the report as a systematic review (title).
ABSTRACT	2b	Provide an explicit statement of the main objective(s) or question(s) the review addresses.
ABSTRACT	2c	Specify the inclusion and exclusion criteria for the review.
ABSTRACT	2d	Specify the information sources (e.g. databases, registers) used to identify studies and the date when each was last searched.
ABSTRACT	2e	Specify the methods used to assess risk of bias in the included studies.
ABSTRACT	2f	Specify the methods used to present and synthesise results.
ABSTRACT	2g	Give the total number of included studies and participants and summarise relevant characteristics of studies.
ABSTRACT	2h	Present results for main outcomes, preferably indicating the number of included studies and participants for each. If meta-analysis was done, report the summary estimate and confidence/credible interval. If comparing groups, indicate the direction of the effect.
ABSTRACT	2i	Provide a brief summary of the limitations of the evidence included in the review (e.g. study risk of bias, inconsistency, imprecision).
ABSTRACT	2j	Provide a general interpretation of the results and important implications.
ABSTRACT	2k	Specify the primary source of funding for the review.
ABSTRACT	2l	Provide the register name and registration number.
INTRODUCTION	3	Describe the rationale for the review in the context of existing knowledge.
INTRODUCTION	4	Provide an explicit statement of the objective(s) or question(s) the review addresses.
METHODS	5	Specify the inclusion and exclusion criteria for the review and how studies were grouped for the syntheses.
METHODS	6	Specify all databases, registers, websites, organisations, reference lists and other sources searched or consulted to identify studies. Specify the date when each source was last searched or consulted.
METHODS	7	Present the full search strategies for all databases, registers and websites, including any filters and limits used.
METHODS	8	Specify the methods used to decide whether a study met the inclusion criteria of the review, including how many reviewers screened each record and each report retrieved, whether they worked independently, and if applicable, details of automation tools used in the process.
METHODS	9	Specify the methods used to collect data from reports, including how many reviewers collected data, whether they worked independently, and if applicable, details of automation tools used.
METHODS	10a	List and define all outcomes for which data were sought. Specify whether all compatible results were sought, and if not, the methods used to decide which results to collect.
METHODS	10b	List and define all other variables for which data were sought (e.g. participant and intervention characteristics, funding sources). Describe any assumptions made about missing or unclear information.
METHODS	11	Specify the methods used to assess risk of bias in the included studies, including details of the tool(s) used, how many reviewers assessed each study and whether they worked independently, and if applicable, details of automation tools used.
METHODS	12	Specify for each outcome the effect measure(s) (e.g. risk ratio, mean difference) used in the synthesis or presentation of results.
METHODS	13a	Describe the processes used to decide which studies were eligible for each synthesis.
METHODS	13b	Describe any methods required to prepare the data for presentation or synthesis.
METHODS	13c	Describe any methods used to tabulate or visually display results of individual studies and syntheses.

METHODS	13d	Describe any methods used to synthesize results and provide a rationale for the choice(s). If meta-analysis was performed, describe the model(s), method(s), heterogeneity assessments, and software used.
METHODS	13e	Describe any methods used to explore possible causes of heterogeneity among study results.
METHODS	13f	Describe any sensitivity analyses conducted to assess robustness of the synthesized results.
METHODS	14	Describe any methods used to assess risk of bias due to missing results in a synthesis (reporting biases).
METHODS	15	Describe any methods used to assess certainty (or confidence) in the body of evidence for an outcome.
RESULTS	16a	Describe the results of the search and selection process, ideally using a flow diagram.
RESULTS	16b	Cite studies that might appear to meet the inclusion criteria, but which were excluded, and explain why.
RESULTS	17	Cite each included study and present its characteristics.
RESULTS	18	Present assessments of risk of bias for each included study.
RESULTS	19	For all outcomes, present summary statistics for each group and an effect estimate with its precision.
RESULTS	20a	For each synthesis, briefly summarise the characteristics and risk of bias among contributing studies.
RESULTS	20b	Present results of all statistical syntheses conducted.
RESULTS	20c	Present results of all investigations of possible causes of heterogeneity among study results.
RESULTS	20d	Present results of all sensitivity analyses conducted to assess robustness of the synthesized results.
RESULTS	21	Present assessments of risk of bias due to missing results (reporting biases) for each synthesis assessed.
RESULTS	22	Present assessments of certainty (or confidence) in the body of evidence for each outcome assessed.
DISCUSSION	23a	Provide a general interpretation of the results in the context of other evidence.
DISCUSSION	23b	Discuss any limitations of the evidence included in the review.
DISCUSSION	23c	Discuss any limitations of the review processes used.
DISCUSSION	23d	Discuss implications of the results for practice, policy, and future research.
OTHER INFORMATION	24a	Provide registration information for the review, including register name and registration number, or state that the review was not registered.
OTHER INFORMATION	24b	Indicate where the review protocol can be accessed, or state that a protocol was not prepared.
OTHER INFORMATION	24c	Describe and explain any amendments to information provided at registration or in the protocol.
OTHER INFORMATION	25	Describe sources of financial or non-financial support for the review, and the role of funders or sponsors.
OTHER INFORMATION	26	Declare any competing interests of review authors.
OTHER INFORMATION	27	Report which materials are publicly available (e.g. data collection forms, extracted data, analytic code, other materials) and where they can be found.

Supplemental Materials 7: *LLM-as-a-Judge Prompts and R Code*

```[r]
# LLM-as-a-Judge section-specific PRISMA prompts
checklist_by_section = list( title = c("Item 1 Title: Does the title identify the report as a systematic
review/meta-analysis? Elements: 1) Identify the report as a systematic review in the title. 2) Report an
informative title that provides key information about the main objective or question the review
addresses (e.g. the population(s) and intervention(s) the review addresses). 3) Consider providing
additional information in the title, such as the method of analysis used, the designs of included studies,
or an indication that the review is an update of an existing review, or a continually updated (“living”)
systematic review."),
abstract = c("Item 2a Title - Title: Does the abstract section have a title that identifies the report as a
systematic review?",
"Item 2b Background - Objectives: Does the abstract section provide an explicit statement of the main
objective(s) or question(s) the review addresses?",
"Item 2c Methods - Eligibility Criteria: Does the abstract section specify the inclusion and exclusion
criteria for the review?",
"Item 2d Methods - Information Sources: Does the abstract section specify the information sources (e.g.
databases, registers) used to identify studies and the date when each was last searched?",
"Item 2e Methods - Risk of Bias: Does the abstract section specify the methods used to assess risk of bias
in the included studies?",
"Item 2f Methods - Synthesis of Results: Does the abstract section specify the methods used to present
and synthesize results?",
"Item 2g Results - Included Studies: Does the abstract section give the total number of included studies
and participants and summarise relevant characteristics of studies?",
"Item 2h Results - Synthesis of Results: Does the abstract section present results for main outcomes,
preferably indicating the number of included studies and participants for each. If meta-analysis was
done, report the summary estimate and confidence/credible interval. If comparing groups, indicate the
direction of the effect (i.e. which group is favoured)?",
"Item 2i Discussion - Limitations of Evidence: Does the abstract section provide a brief summary of the
limitations of the evidence included in the review (e.g. study risk of bias, inconsistency and
imprecision)?",
"Item 2j Discussion - Interpretation: Does the abstract section provide a general interpretation of the
results and important implications?",
"Item 2k Other - Funding: Does the abstract section specify the primary source of funding for the
review?",
"Item 2l Other - Registration: Does the abstract section provide the register name and registration
number?"),
intro = c("Item 3 Rationale: Does the intro describe the rationale for the review in the context of existing
knowledge? Elements: 1) Describe the current state of knowledge and its uncertainties. 2) Articulate why
it is important to do the review. 3) If other systematic reviews addressing the same (or a largely similar)
question are available, explain why the current review was considered necessary. If the review is an
update or replication of a particular systematic review, indicate this and cite the previous review. 4) If the
review examines the effects of interventions, also briefly describe how the intervention(s) examined
might work. 5) If there is complexity in the intervention or context of its delivery (or both) (e.g. multi-
component interventions, equity considerations), consider presenting a logic model to visually display
the hypothesised relationship between intervention components and outcomes.",
"Item 4 Objectives: Does the intro provide an explicit statement of the objective(s) or question(s) the
review addresses. Elements: 1) Provide an explicit statement of all objective(s) or question(s) the review
```

addresses, expressed in terms of a relevant question formulation framework. 2) If the purpose is to evaluate the effects of interventions, use the Population, Intervention, Comparator, Outcome (PICO) framework or one of its variants, to state the comparisons that will be made."),
methods = c("Item 5 Eligibility Criteria: Does the methods section specify the inclusion and exclusion criteria for the review and how studies were grouped for the syntheses. Elements: 1) Specify all study characteristics used to decide whether a study was eligible for inclusion in the review, that is, components described in the PICO framework or one of its variants, and other characteristics, such as eligible study design(s) and setting(s), and minimum duration of follow-up. 2) Specify eligibility criteria with regard to report characteristics, such as year of dissemination, language, and report status (e.g. whether reports, such as unpublished manuscripts and conference abstracts, were eligible for inclusion). 3) Clearly indicate if studies were ineligible because the outcomes of interest were not measured, or ineligible because the results for the outcome of interest were not reported. 4) Specify any groups used in the synthesis (e.g. intervention, outcome and population groups) and link these to the comparisons specified in the objectives (Item 4). 5) Consider providing rationales for any notable restrictions to study eligibility.",
"Item 6 Information Sources: Does the methods section specify all databases, registers, websites, organisations, reference lists and other sources searched or consulted to identify studies. Specify the date when each source was last searched or consulted. Elements: 1) Specify the date when each source (e.g. database, register, website, organisation) was last searched or consulted. 2) If bibliographic databases were searched, specify for each database its name (e.g. MEDLINE, CINAHL), the interface or platform through which the database was searched (e.g. Ovid, EBSCOhost), and the dates of coverage (where this information is provided). 3) If study registers, regulatory databases and other online repositories were searched, specify the name of each source and any date restrictions that were applied. 4) If websites, search engines or other online sources were browsed or searched, specify the name and URL of each source. 5) If organisations or manufacturers were contacted to identify studies, specify the name of each source. 6) If individuals were contacted to identify studies, specify the types of individuals contacted (e.g. authors of studies included in the review or researchers with expertise in the area). 7) If reference lists were examined, specify the types of references examined (e.g. references cited in study reports included in the systematic review, or references cited in systematic review reports on the same or similar topic). 8) If cited or citing reference searches (also called backward and forward citation searching) were conducted, specify the bibliographic details of the reports to which citation searching was applied, the citation index or platform used (e.g. Web of Science), and the date the citation searching was done. 9) If journals or conference proceedings were consulted, specify of the names of each source, the dates covered and how they were searched (e.g. handsearching or browsing online).",
"Item 7 Search Strategy: Does the methods section present the full search strategies for all databases, registers and websites, including any filters and limits used. Element: 1) Provide the full line by line search strategy as run in each database with a sophisticated interface (such as Ovid), or the sequence of terms that were used to search simpler interfaces, such as search engines or websites. 2) Describe any limits applied to the search strategy (e.g. date or language) and justify these by linking back to the review’s eligibility criteria. 3) If published approaches, including search filters designed to retrieve specific types of records or search strategies from other systematic reviews, were used, cite them. If published approaches were adapted, for example if search filters are amended, note the changes made. 4) If natural language processing or text frequency analysis tools were used to identify or refine keywords, synonyms or subject indexing terms to use in the search strategy, specify the tool(s) used. 5) If a tool was used to automatically translate search strings for one database to another, specify the tool used. 6) If the search strategy was validated, for example by evaluating whether it could identify a set of clearly eligible studies, report the validation process used and specify which studies were included in the validation set. 7) If the search strategy was peer reviewed, report the peer review process used and

specify any tool used such as the Peer Review of Electronic Search Strategies (PRESS) checklist. 8) If the search strategy structure adopted was not based on a PICO-style approach, describe the final conceptual structure and any explorations that were undertaken to achieve it.", "Item 8 Selection Process: Does the methods section specify the methods used to decide whether a study met the inclusion criteria of the review, including how many reviewers screened each record and each report retrieved, whether they worked independently, and if applicable, details of automation tools used in the process. Elements: 1) Recommendations for reporting regardless of the selection processes used: 1A) Report how many reviewers screened each record (title/abstract) and each report retrieved, whether multiple reviewers worked independently at each stage of screening or not, and any processes used to resolve disagreements between screeners. 1B) Report any processes used to obtain or confirm relevant information from study investigators. 1C) If abstracts or articles required translation into another language to determine their eligibility, report how these were translated. 2) Recommendations for reporting in systematic reviews using automation tools in the selection process: 2A) Report how automation tools were integrated within the overall study selection process. 2B) If an externally derived machine learning classifier was applied (e.g. Cochrane RCT Classifier), either to eliminate records or to replace a single screener, include a reference or URL to the version used. If the classifier was used to eliminate records before screening, report the number eliminated in the PRISMA flow diagram as 'Records marked as ineligible by automation tools'. 2C) If an internally derived machine learning classifier was used to assist with the screening process, identify the software/classifier and version, describe how it was used (e.g. to remove records or replace a single screener) and trained (if relevant), and what internal or external validation was done to understand the risk of missed studies or incorrect classifications. 2D) If machine learning algorithms were used to prioritise screening (whereby unscreened records are continually re-ordered based on screening decisions), state the software used and provide details of any screening rules applied. 3) Recommendations for reporting in systematic reviews using crowdsourcing or previous 'known' assessments in the selection process: 3A) If crowdsourcing was used to screen records, provide details of the platform used and specify how it was integrated within the overall study selection process. 3B) If datasets of already-screened records were used to eliminate records retrieved by the search from further consideration, briefly describe the derivation of these datasets.",

"Item 9 Data Collection Process: Does the methods section specify the methods used to collect data from reports, including how many reviewers collected data from each report, whether they worked independently, any processes for obtaining or confirming data from study investigators, and if applicable, details of automation tools used in the process. Elements: 1) Report how many reviewers collected data from each report, whether multiple reviewers worked independently or not, and any processes used to resolve disagreements between data collectors. 2) Report any processes used to obtain or confirm relevant data from study investigators. 3) If any automation tools were used to collect data, report how the tool was used, how the tool was trained, and what internal or external validation was done to understand the risk of incorrect extractions. 4) If articles required translation into another language to enable data collection, report how these articles were translated. 5) If any software was used to extract data from figures, specify the software used. 6) If any decision rules were used to select data from multiple reports corresponding to a study, and any steps were taken to resolve inconsistencies across reports, report the rules and steps used.",

"Item 10a Data Items (outcomes): Does the methods section list and define all outcomes for which data were sought. Specify whether all results that were compatible with each outcome domain in each study were sought (e.g. for all measures, time points, analyses), and if not, the methods used to decide which results to collect. Elements: 1) List and define the outcome domains and time frame of measurement for which data were sought. 2) Specify whether all results that were compatible with each outcome domain in each study were sought, and if not, what process was used to select results within eligible domains. 3)

If any changes were made to the inclusion or definition of the outcome domains, or to the importance given to them in the review, specify the changes, along with a rationale. 4) If any changes were made to the processes used to select results within eligible outcome domains, specify the changes, along with a rationale. 5) Consider specifying which outcome domains were considered the most important for interpreting the review's conclusions and provide rationale for the labelling (e.g. "a recent core outcome set identified the outcomes labelled 'critical' as being the most important to patients").",
"Item 10b Data Items (other variables): Does the methods section list and define all other variables for which data were sought (e.g. participant and intervention characteristics, funding sources). Describe any assumptions made about any missing or unclear information. Elements: 1) List and define all other variables for which data were sought (e.g. participant and intervention characteristics, funding sources). 2) Describe any assumptions made about any missing or unclear information from the studies. 3) If a tool was used to inform which data items to collect, cite the tool used.",
"Item 11 Study Risk of Bias Assessment: Does the methods section specify the methods used to assess risk of bias in the included studies, including details of the tool(s) used, how many reviewers assessed each study and whether they worked independently, and if applicable, details of automation tools used in the process. Elements: 1) Specify the tool(s) (and version) used to assess risk of bias in the included studies. 2) Specify the methodological domains/components/items of the risk of bias tool(s) used. 3) Report whether an overall risk of bias judgement that summarised across domains/components/items was made, and if so, what rules were used to reach an overall judgement. 4) If any adaptations to an existing tool to assess risk of bias in studies were made, specify the adaptations. 5) If a new risk of bias tool was developed for use in the review, describe the content of the tool and make it publicly accessible. 6) Report how many reviewers assessed risk of bias in each study, whether multiple reviewers worked independently, and any processes used to resolve disagreements between assessors. 7) Report any processes used to obtain or confirm relevant information from study investigators. 8) If an automation tool was used to assess risk of bias, report how the automation tool was used, how the tool was trained, and details on the tool's performance and internal validation.",
"Item 12 Effect Measures: Does the methods section specifyfor each outcome the effect measure(s) (e.g. risk ratio, mean difference) used in the synthesis or presentation of results. Elements: 1) Specify for each outcome (or type of outcome [e.g. binary, continuous]), the effect measure(s) (e.g. risk ratio, mean difference) used in the synthesis or presentation of results. 2) State any thresholds (or ranges) used to interpret the size of effect (e.g. minimally important difference; ranges for no/trivial, small, moderate and large effects) and the rationale for these thresholds. 3) If synthesized results were re-expressed to a different effect measure, report the method used to re-express results (e.g. meta-analysing risk ratios and computing an absolute risk reduction based on an assumed comparator risk). 4) Consider providing justification for the choice of effect measure.",
"Item 13a Synthesis Methods (eligibility for synthesis): Does the methods section describe the processes used to decide which studies were eligible for each synthesis (e.g. tabulating the study intervention characteristics and comparing against the planned groups for each synthesis (Item 5)). Element: 1) Describe the processes used to decide which studies were eligible for each synthesis.",
"Item 13b Synthesis Methods (preparing for synthesis): Does the methods section describe any methods required to prepare the data for presentation or synthesis, such as handling of missing summary statistics, or data conversions. Element: 1) Report any methods required to prepare the data collected from studies for presentation or synthesis, such as handling of missing summary statistics, or data conversions.",
"Item 13c Synthesis Methods (tabulation and graphical methods): Does the methods section describe any methods used to tabulate or visually display results of individual studies and syntheses. Elements: 1) Report chosen tabular structure(s) used to display results of individual studies and syntheses, along with details of the data presented. 2) Report chosen graphical methods used to visually display results of

individual studies and syntheses. 3) If studies are ordered or grouped within tables or graphs based on study characteristics (e.g. by size of the study effect, year of publication), consider reporting the basis for the chosen ordering/grouping. 4) If non-standard graphs were used, consider reporting the rationale for selecting the chosen graph.",
"Item 13d Synthesis Methods (statistical synthesis methods): Does the methods section describe any methods used to synthesize results and provide a rationale for the choice(s). If meta-analysis was performed, describe the model(s), method(s) to identify the presence and extent of statistical heterogeneity, and software package(s) used. Elements: 1) If statistical synthesis methods were used, reference the software, packages and version numbers used to implement synthesis methods. 2) If it was not possible to conduct a meta-analysis, describe and justify the synthesis methods or summary approach used. 3) If meta-analysis was done, specify: 3A) the meta-analysis model (fixed-effect, fixed-effects or random-effects) and provide rationale for the selected model. 3B) the method used (e.g. Mantel-Haenszel, inverse-variance). 3C) any methods used to identify or quantify statistical heterogeneity (e.g. visual inspection of results, a formal statistical test for heterogeneity, heterogeneity variance (τ2), inconsistency (e.g. I2), and prediction intervals). 4) If a random-effects meta-analysis model was used: 4A) specify the between-study (heterogeneity) variance estimator used (e.g. DerSimonian and Laird, restricted maximum likelihood (REML)). 4B) specify the method used to calculate the confidence interval for the summary effect (e.g. Wald-type confidence interval, Hartung-Knapp-SidikJonkman). 4C) consider specifying other details about the methods used, such as the method for calculating confidence limits for the heterogeneity variance. 5) If a Bayesian approach to meta-analysis was used, describe the prior distributions about quantities of interest (e.g. intervention effect being analysed, amount of heterogeneity in results across studies). 6) If multiple effect estimates from a study were included in a meta-analysis, describe the method(s) used to model or account for the statistical dependency (e.g. multivariate meta-analysis, multilevel models or robust variance estimation). 7) If a planned synthesis was not considered possible or appropriate, report this and the reason for that decision.",
"Item 13e Synthesis Methods (methods to explore heterogeneity): Does the methods section describe any methods used to explore possible causes of heterogeneity among study results (e.g. subgroup analysis, meta-regression). Elements: 1) If methods were used to explore possible causes of statistical heterogeneity, specify the method used (e.g. subgroup analysis, meta-regression). 2) If subgroup analysis or meta-regression was performed, specify for each: 2A) which factors were explored, levels of those factors, and which direction of effect modification was expected and why (where possible). 2B) whether analyses were conducted using study-level variables (i.e. where each study is included in one subgroup only), within-study contrasts (i.e. where data on subsets of participants within a study are available, allowing the study to be included in more than one subgroup), or some combination of the above. 2C) how subgroup effects were compared (e.g. statistical test for interaction for subgroup analyses). 3) If other methods were used to explore heterogeneity because data were not amenable to meta-analysis of effect estimates (e.g. structuring tables to examine variation in results across studies based on subpopulation), describe the methods used, along with the factors and levels. 4) If any analyses used to explore heterogeneity were not pre-specified, identify them as such.",
"Item 13f Synthesis Methods (sensitivity analyses): Does the methods section describe any sensitivity analyses conducted to assess robustness of the synthesized results. Elements: 1) If sensitivity analyses were performed, provide details of each analysis (e.g. removal of studies at high risk of bias, use of an alternative meta-analysis model). 2) If any sensitivity analyses were not pre-specified, identify them as such.",
"Item 14 Reporting Bias Assessment: Does the methods section describe any methods used to assess risk of bias due to missing results in a synthesis (arising from reporting biases). Elements: 1) Specify the methods (tool, graphical, statistical or other) used to assess the risk of bias due to missing results in a

synthesis (arising from reporting biases). 2) If risk of bias due to missing results was assessed using an existing tool, specify the methodological components/domains/items of the tool, and the process used to reach a judgement of overall risk of bias. 3) If any adaptations to an existing tool to assess risk of bias due to missing results were made, specify the adaptations. 4) If a new tool to assess risk of bias due to missing results was developed for use in the review, describe the content of the tool and make it publicly accessible. 5) Report how many reviewers assessed risk of bias due to missing results in a synthesis, whether multiple reviewers worked independently, and any processes used to resolve disagreements between assessors. 6) Report any processes used to obtain or confirm relevant information from study investigators. 7) If an automation tool was used to assess risk of bias due to missing results, report how the automation tool was used, how the tool was trained, and details on the tool's performance and internal validation.",
"Item 15 Certainty Assessment: Does the methods section describe any methods used to assess certainty (or confidence) in the body of evidence for an outcome. Elements: 1) Specify the tool or system (and version) used to assess certainty (or confidence) in the body of evidence. 2) Report the factors considered (e.g. precision of the effect estimate, consistency of findings across studies) and the criteria used to assess each factor when assessing certainty in the body of evidence. 3) Describe the decision rules used to arrive at an overall judgement of the level of certainty, together with the intended interpretation (or definition) of each level of certainty. 4) If applicable, report any review-specific considerations for assessing certainty, such as thresholds used to assess imprecision and ranges of magnitude of effect that might be considered trivial, moderate or large, and the rationale for these thresholds and ranges (Item 12). 5) If any adaptations to an existing tool or system to assess certainty were made, specify the adaptations. 6) Report how many reviewers assessed certainty in the body of evidence for an outcome, whether multiple reviewers worked independently, and any processes used to resolve disagreements between assessors. 7) Report any processes used to obtain or confirm relevant information from investigators. 8) If an automation tool was used to support the assessment of certainty, report how the automation tool was used, how the tool was trained, and details on the tool's performance and internal validation. 9) Describe methods for reporting the results of assessments of certainty, such as the use of Summary of Findings tables. 10) If standard phrases that incorporate the certainty of evidence were used (e.g. "hip protectors probably reduce the risk of hip fracture slightly"), report the intended interpretation of each phrase and the reference for the source guidance."),
results = c("Item 16a Study Selection (flow of studies): Does the results section describe the results of the search and selection process, from the number of records identified in the search to the number of studies included in the review, ideally using a flow diagram. Elements: 1) Report, ideally using a flow diagram, the number of: records identified; records excluded before screening; records screened; records excluded after screening titles or titles and abstracts; reports retrieved for detailed evaluation; potentially eligible reports that were not retrievable; retrieved reports that did not meet inclusion criteria and the primary reasons for exclusion; and the number of studies and reports included in the review. If applicable, also report the number of ongoing studies and associated reports identified. 2) If the review is an update of a previous review, report results of the search and selection process for the current review and specify the number of studies included in the previous review. 3) If applicable, indicate in the PRISMA flow diagram how many records were excluded by a human and how many by automation tools.",
"Item 16b Study Selection (excluded studies): Does the results section cite studies that might appear to meet the inclusion criteria, but which were excluded, and explain why they were excluded. Element: 1) Cite studies that might appear to meet the inclusion criteria, but which were excluded, and explain why they were excluded.",
"Item 17 Study Characteristics: Does the results section cite each included study and present its characteristics. Elements: 1) Cite each included study. 2) Present the key characteristics of each study in a

table or figure (considering a format that will facilitate comparison of characteristics across the studies). 3) If the review examines the effects of interventions, consider presenting an additional table that summarises the intervention details for each study.",
"Item 18 Risk of Bias in Studies: Does the results section present assessments of risk of bias for each included study. Elements: 1) Present tables or figures indicating for each study the risk of bias in each domain/component/item assessed (e.g. blinding of outcome assessors, missing outcome data) and overall study-level risk of bias. 2) Present justification for each risk of bias judgement, for example in the form of relevant quotations from reports of included studies. 3) If assessments of risk of bias were done for specific outcomes or results in each study, consider displaying risk of bias judgements on a forest plot, next to the study results.",
"Item 19 Results of Individual Studies: Does the results section, for all outcomes, present, for each study: (a) summary statistics for each group (where appropriate) and (b) an effect estimate and its precision (e.g. confidence/credible interval), ideally using structured tables or plots. Elements: 1) For all outcomes, irrespective of whether statistical synthesis was undertaken, present for each study summary statistics for each group (where appropriate). For dichotomous outcomes, report the number of participants with and without the events for each group; or the number with the event and the total for each group (e.g. 12/45). For continuous outcomes, report the mean, standard deviation and sample size of each group. 2) For all outcomes, irrespective of whether statistical synthesis was undertaken, present for each study an effect estimate and its precision (e.g. standard error or 95% confidence/credible interval). For example, for time-to-event outcomes, present a hazard ratio and its confidence interval. 3) If study-level data is presented visually or reported in the text (or both), also present a tabular display of the results. 4) If results were obtained from multiple data sources (e.g. journal article, study register entry, clinical study report, correspondence with authors), report the source of the data. 5) If applicable, indicate which results were not reported directly and had to be computed or estimated from other information.",
"Item 20a Results of Syntheses (characteristics of contributing studies): Does the results section, for each synthesis, briefly summarise the characteristics and risk of bias among contributing studies. Elements: 1) Provide a brief summary of the characteristics and risk of bias among studies contributing to each synthesis (meta-analysis or other). The summary should focus only on study characteristics that help in interpreting the results (especially those that suggest the evidence addresses only a restricted part of the review question, or indirectly addresses the question). 2) Indicate which studies were included in each synthesis (e.g. by listing each study in a forest plot or table or citing studies in the text).",
"Item 20b Results of Syntheses (results of statistical syntheses): Does the results section present results of all statistical syntheses conducted. If meta-analysis was done, present for each the summary estimate and its precision (e.g. confidence/credible interval) and measures of statistical heterogeneity. If comparing groups, describe the direction of the effect. Elements: 1) Report results of all statistical syntheses described in the protocol and all syntheses conducted that were not pre-specified. 2) If meta-analysis was conducted, report for each: 2A) the summary estimate and its precision (e.g. standard error or 95% confidence/credible interval) 2B) measures of statistical heterogeneity (e.g. τ2, I2, prediction interval) 3) If other statistical synthesis methods were used (e.g. summarising effect estimates, combining P values), report the synthesized result and a measure of precision (or equivalent information, for example, the number of studies and total sample size). 4) If the statistical synthesis method does not yield an estimate of effect (e.g. as is the case when P values are combined), report the relevant statistics (e.g. P value from the statistical test), along with an interpretation of the result that is consistent with the question addressed by the synthesis method. 5) If comparing groups, describe the direction of effect (e.g. fewer events in the intervention group, or higher pain in the comparator group). 6) If synthesising mean differences, specify for each synthesis, where applicable, the unit of measurement (e.g. kilograms or pounds for weight), the upper and lower limits of the measurement scale (e.g. anchors range from 0 to 10), direction of benefit (e.g. higher scores denote higher severity of pain), and the minimally

important difference, if known. If synthesising standardised mean differences, and the effect estimate is being re-expressed to a particular instrument, details of the instrument, as per the mean difference, should be reported.",
"Item 20c Results of Syntheses (results of investigations of heterogeneity): Does the results section present results of all investigations of possible causes of heterogeneity among study results. Elements: 1) If investigations of possible causes of heterogeneity were conducted: 1A) present results regardless of the statistical significance, magnitude, or direction of effect modification. 1B) identify the studies contributing to each subgroup. 1C) report results with due consideration to the observational nature of the analysis and risk of confounding due to other factors. 2) If subgroup analysis was conducted: 2A) report for each analysis the exact P value for a test for interaction, as well as, within each subgroup, the summary estimates, their precision (e.g. standard error or 95% confidence/credible interval) and measures of heterogeneity. 2B) consider presenting the estimate for the difference between subgroups and its precision. 3) If meta-regression was conducted: 3A) report for each analysis the exact P value for the regression coefficient and its precision. 3B) consider presenting a meta-regression scatterplot with the study effect estimates plotted against the potential effect modifier. 4) If informal methods (i.e. those that do not involve a formal statistical test) were used to investigate heterogeneity, describe the results observed.",
"Item 20d Results of Syntheses (results of sensitivity analyses): Does the results section present results of all sensitivity analyses conducted to assess the robustness of the synthesized results. Elements: 1) If any sensitivity analyses were conducted: 1A) report the results for each sensitivity analysis. 1B) comment on how robust the main analysis was given the results of all corresponding sensitivity analyses. 1C) consider presenting results in tables that indicate: (i) the summary effect estimate, a measure of precision (and potentially other relevant statistics, for example, I2 statistic) and contributing studies for the original meta-analysis; (ii) the same information for the sensitivity analysis; and (iii) details of the original and sensitivity analysis assumptions. 1D) consider presenting results of sensitivity analyses visually using forest plots.",
"Item 21 Reporting Biases: Does the results section present assessments of risk of bias due to missing results (arising from reporting biases) for each synthesis assessed. Elements: 1) Present assessments of risk of bias due to missing results (arising from reporting biases) for each synthesis assessed. 2) If a tool was used to assess risk of bias due to missing results in a synthesis, present responses to questions in the tool, judgements about risk of bias and any information used to support such judgements. 3) If a funnel plot was generated to evaluate small-study effects (one cause of which is reporting biases), present the plot and specify the effect estimate and measure of precision used in the plot. If a contour-enhanced funnel plot was generated, specify the 'milestones' of statistical significance that the plotted contour lines represent (P = 0.01, 0.05, 0.1, etc.) 4) If a test for funnel plot asymmetry was used, report the exact P value observed for the test, and potentially other relevant statistics, for example the standardised normal deviate, from which the P value is derived. 5) If any sensitivity analyses seeking to explore the potential impact of missing results on the synthesis were conducted, present results of each analysis (see item #20d), compare them with results of the primary analysis, and report results with due consideration of the limitations of the statistical method. 6) If studies were assessed for selective non-reporting of results by comparing outcomes and analyses pre-specified in study registers, protocols, and statistical analysis plans with results that were available in study reports, consider presenting a matrix (with rows as studies and columns as syntheses) to present the availability of study results. 7) If an assessment of selective non-reporting of results reveals that some studies are missing from the synthesis, consider displaying the studies with missing results underneath a forest plot or including a table with the available study results.",
"Item 22 Certainty of Evidence: Does the results section present assessments of certainty (or confidence) in the body of evidence for each outcome assessed. Elements: 1) Report the overall level of certainty (or

confidence) in the body of evidence for each important outcome. 2) Provide an explanation of reasons for rating down (or rating up) the certainty of evidence (e.g. in footnotes to an evidence summary table). 3) Communicate certainty in the evidence wherever results are reported (i.e. abstract, evidence summary tables, results, conclusions), using a format appropriate for the section of the review. 4) Consider including evidence summary tables, such as GRADE Summary of Findings tables."),
discussion = c("Item 23a Discussion (interpretation): Does the discussion section provide a general interpretation of the results in the context of other evidence. Element: 1) Provide a general interpretation of the results in the context of other evidence.",
"Item 23b Discussion (limitations of evidence): Does the discussion section discuss any limitations of the evidence included in the review. Element: 1) Discuss any limitations of the evidence included in the review.",
"Item 23c Discussion (limitations of review processes): Does the discussion section discuss any limitations of the review processes used. Element: 1) Discuss any limitations of the review processes used, and comment on the potential impact of each limitation.",
"Item 23d Discussion (implications): Does the discussion section discuss implications of the results for practice, policy, and future research. Elements: 1) Discuss implications of the results for practice and policy. 2) Make explicit recommendations for future research."),
other = c("Item 24a Registration and Protocol (registration): Does the other section provide registration information for the review, including register name and registration number, or state that the review was not registered. Element: 1) Provide registration information for the review, including register name and registration number, or state that the review was not registered.",
"Item 24b Registration and Protocol (protocol): Does the other section indicate where the review protocol can be accessed, or state that a protocol was not prepared. Element: 1) Indicate where the review protocol can be accessed (e.g. by providing a citation, DOI or link), or state that a protocol was not prepared.",
"Item 24c Registration and Protocol (amendments): Does the other section describe and explain any amendments to information provided at registration or in the protocol. Element: 1) Report details of any amendments to information provided at registration or in the protocol, noting: (a) the amendment itself; (b) the reason for the amendment; and (c) the stage of the review process at which the amendment was implemented.",
"Item 25 Support: Does the other section describe sources of financial or non-financial support for the review, and the role of the funders or sponsors in the review. Elements: 1) Describe sources of financial or non-financial support for the review, specifying relevant grant ID numbers for each funder. If no specific financial or nonfinancial support was received, this should be stated. 2) Describe the role of the funders or sponsors (or both) in the review. If funders or sponsors had no role in the review, this should be declared.",
"Item 26 Competing Interests: Does the other section declare any competing interests of review authors. Elements: 1) Disclose any of the authors' relationships or activities that readers could consider pertinent or to have influenced the review. 2) If any authors had competing interests, report how they were managed for particular review processes.",
"Item 27 Availability of Data, Code, and Other Materials: Does the other section report which of the following are publicly available and where they can be found: template data collection forms; data extracted from included studies; data used for all analyses; analytic code; any other materials used in the review. Elements: 1) Report which of the following are publicly available: template data collection forms; data extracted from included studies; data used for all analyses; analytic code; any other materials used in the review. 2) If any of the above materials are publicly available, report where they can be found (e.g. provide a link to files deposited in a public repository). 3) If data, analytic code, or other materials will be

```
made available upon request, provide the contact details of the author responsible for sharing the
materials and describe the circumstances under which such materials will be shared.") )
max_score =length(checklist_by_section$title) + length(checklist_by_section$abstract) +
length(checklist_by_section$intro) + length(checklist_by_section$methods) +
length(checklist_by_section$results) + length(checklist_by_section$discussion) +
length(checklist_by_section$other)
names(checklist_by_section$title) = c("Item 1") names(checklist_by_section$abstract) = c("Item 2a",
"Item 2b", "Item 2c", "Item 2d", "Item 2e", "Item 2f", "Item 2g", "Item 2h", "Item 2i", "Item 2j", "Item
2k", "Item 2l") names(checklist_by_section$intro) = c("Item 3", "Item 4")
names(checklist_by_section$methods) = c("Item 5", "Item 6", "Item 7", "Item 8", "Item 9", "Item 10a",
"Item 10b", "Item 11", "Item 12", "Item 13a", "Item 13b", "Item 13c", "Item 13d", "Item 13e", "Item
13f", "Item 14", "Item 15") names(checklist_by_section$results) = c("Item 16a", "Item 16b", "Item 17",
"Item 18", "Item 19", "Item 20a", "Item 20b", "Item 20c", "Item 20d", "Item 21", "Item 22")
names(checklist_by_section$discussion) = c("Item 23a", "Item 23b", "Item 23c", "Item 23d")
names(checklist_by_section$other) = c("Item 24a", "Item 24b", "Item 24c", "Item 25", "Item 26", "Item
27")
#Define function to build a section-specific prompt
make_section_prompt <- function(section_name, checklist_items) { paste0( "You are a systematic
reviewer evaluating the '", section_name, "' section of a manuscript using the PRISMA 2020
checklist.\n\n", "Each section has an item and elements of that item that must be satisfied. Here are the
checklist items for this section:\n\n", paste(checklist_items, collapse = "\n"), "\n\n", "Return a JSON
object where each key is a checklist item (e.g., 'Item 1'), and each value is an object with the following
fields:
"manuscript": the name of the manuscript (e.g., "o1_mini_reasoning_paper_1").
"item": the checklist item identifier (e.g., "Item 1").
"score": a continuous value between 0-1 (inclusive of both 0 and 1). 0 means the section is non-
compliant and 1 means the section is compliant with the checklist item and the associated elements.
"rationale": a concise explanation of the score.
"citation": a quote or quotes from the section that supports the rationale.
Return only the JSON object, no extra commentary or formatting.\n\n",
manuscript_vector[[section_name]] ) }
```

<u>Supplemental Material 8: Prompt Engineering Strategy for LLM-Assisted Literature Screening</u>

The prompt engineering strategy employed in this study followed an iterative, human-in-the-loop development process designed to maximize screening sensitivity while maintaining acceptable specificity. The strategy was applied independently for each of the two clinical topics (carvedilol in compensated cirrhosis and anticoagulation in portal vein thrombosis). The core principles guiding the process were:

1. Transparency and reproducibility of inclusion/exclusion logic,
2. Provision of concrete examples to reduce ambiguity,
3. Emphasis on edge cases identified during pilot testing, and
4. Structured output formatting to facilitate downstream processing.

The development of each screening prompt proceeded through four stages.

- <u>Stage 1 (Initial Prompt Construction)</u> – A baseline prompt was drafted that included the study's inclusion and exclusion criteria translated into natural language instructions. The prompt specified the target population, intervention/exposure, comparator, and outcomes of interest (PICO framework). The LLM was instructed to output a binary decision (0 = rule out, 1 = rule in) followed by a brief rationale.
- <u>Stage 2 (Pilot Testing)</u> – The initial prompt was applied to a randomly selected pilot set of 100 deduplicated abstracts per topic. The LLM's decisions were compared against independent human review (gold standard). Performance metrics (sensitivity, specificity, false-positive rate, and false-negative rate) were calculated. Error cases were categorized by type (e.g., missed subgroup relevance, overly strict population interpretation, keyword-driven false positives).
- <u>Stage 3 (Error Analysis and Prompt Refinement)</u> – Misclassified abstracts were reviewed to identify systematic error patterns. The prompt was revised iteratively to address each pattern. Common refinements included adding clarifying notes for ambiguous criteria (e.g., defining decompensation events), inserting emphasis markers (e.g., bold text, asterisks) for critical instructions the LLM had previously overlooked, adding curated examples of correctly ruled-in and ruled-out articles (few-shot prompting), and specifying handling of edge cases (e.g., studies with mixed compensated/decompensated populations, studies reporting zero bleeding events).
- <u>Stage 4 (Validation)</u> – Once the prompt achieved the prespecified performance thresholds (≥90% sensitivity, ≥80% specificity) on the pilot set, it was frozen and applied to a held-out validation set of 100 randomly selected abstracts. Performance was re-evaluated to confirm stability. The finalized prompt was then deployed for full-scale screening of all remaining records.

The following optimization techniques were applied during prompt refinement:

- <u>Few-Shot Prompting</u> – Curated examples of 4-6 articles (both ruled-in and ruled-out) were embedded directly in the prompt. Each example included the article's title and abstract followed by the correct decision and an explicit rationale. Examples were selected to represent common edge cases (e.g., animal studies, review articles, studies with relevant keywords but wrong population). This technique anchored the LLM's decision-making in concrete precedents. See **Supplemental Materials 3 and 4** for the final few-shot examples used.
- <u>Explicit Criteria Embedding</u> – Inclusion and exclusion criteria were presented in structured, bullet-point form within the prompt. Each criterion was stated as a clear, testable condition. This reduced the risk of the LLM applying its own interpretation of clinical relevance rather than adhering to the study-specific criteria.
- <u>Emphasis Markers</u> – Critical instructions that the LLM had overlooked in earlier iterations were highlighted using bold text, asterisks, and explicit labels. For example, instructions regarding the

inclusion of studies reporting zero bleeding events were given special emphasis after the pilot revealed that these were being incorrectly excluded.

- Edge-Case Clarifications – Numbered clarification notes were appended to the prompt to address specific ambiguities identified during error analysis. These included handling of studies with mixed beta-blocker groups (rule in if carvedilol subgroup exists), interpretation of Child-Pugh class A/B populations (cannot determine compensated vs. decompensated), treatment of conference abstracts (rule out), and handling of studies where carvedilol appeared in both treatment and control arms (rule out).
- Structured Output Format – The LLM was instructed to produce a strictly formatted two-line output: (1) a binary decision ("0" or "1"), and (2) a one-sentence rationale. This structured format facilitated automated parsing for downstream analysis and ensured that the LLM committed to a definitive decision before providing its reasoning.

Example: Iterative Prompt Refinement for Carvedilol Screening

The following example illustrates how the screening prompt for the carvedilol in compensated cirrhosis topic was refined through iterative error analysis.

1. Iteration 1 (Baseline Prompt) – The initial prompt included the basic PICO criteria: population (compensated cirrhosis), intervention (carvedilol), comparator (no treatment, placebo, or variceal ligation), and outcomes (decompensation or mortality). The prompt achieved 78% sensitivity and 92% specificity on the pilot set.
2. Error Pattern Identified – Three relevant studies reporting zero variceal bleeding events (e.g., "No patient bled from varices in either group") were incorrectly ruled out. The LLM interpreted the absence of bleeding events as the study not addressing variceal bleeding as an outcome.
3. Iteration 2 (Refinement) – The following clarification was added to the prompt, with emphasis markers: "***Very important point***: Any mention of variceal bleeding in the result, even if no bleeding occurred, should be considered relevant for inclusion. Studies reporting no bleeding events should still be considered if they mention variceal bleeding as a result." A corresponding few-shot example was also added (**Example 6 in Supplemental Material 3**), showing a study with zero bleeding events that should be ruled in. After this refinement, the prompt achieved 92% sensitivity and 90% specificity.
4. Iteration 3 (Final Refinement) – Additional clarifications were added to handle: (a) studies where carvedilol was used alongside other beta-blockers with subgroup analyses, (b) studies mentioning only "cirrhosis" without specifying compensated status (rule in for full-text screening), and (c) studies with Child-Pugh A/B populations where compensation status was ambiguous (rule in). These refinements yielded the final prompt (**Supplemental Material 3**), which achieved >90% sensitivity and >80% specificity on both the pilot and held-out validation sets.

The final screening prompts used for both clinical topics are provided in their entirety in **Supplemental Materials 3 and 4**. The manuscript generation prompt is provided in **Supplemental Material 5**, and the LLM-as-a-Judge evaluation prompts and associated R code are provided in **Supplemental Material 7**. All analysis code is publicly available at https://github.com/YZ-334455/PCORI_Meta_LLM.git.

Researchers seeking to replicate or adapt this approach should note that: (1) LLM outputs are inherently stochastic, so exact numerical reproducibility is not guaranteed across runs or model versions; (2) the iterative prompt refinement process is topic-specific and should be repeated with a new pilot set when applying the methodology to a different clinical question; and (3) a temperature setting of 0 (or as low as available) was used during screening to maximize consistency.
